# Chloroplast genomes of five *Oedogonium* species: genome structure, phylogenetic analysis and adaptive evolution

**DOI:** 10.1186/s12864-021-08006-1

**Published:** 2021-09-30

**Authors:** Qian Xiong, Yuxin Hu, Wenqi Lv, Qinghua Wang, Guoxiang Liu, Zhengyu Hu

**Affiliations:** 1 Institute of Hydrobiology, Chinese Academy of Sciences, Wuhan, 430072 China; 2 University of Chinese Academy of Sciences, Beijing, 100039 China; 3 Yangtze River Basin Ecological Environment Monitoring and Scientific Research Center, Yangtze River Basin Ecological Environment Supervision and Administration Bureau, Ministry of Ecological Environment, Wuhan, China; 4 Department of Biology, Taiyuan Normal University, Jinzhong, 030619 PR China; 5 State Key Laboratory of Freshwater Ecology and Biotechnology, Institute of Hydrobiology, Chinese Academy of Sciences, Wuhan, 430072 China

**Keywords:** Oedogoniales, Chloroplast genome, Synteny, Polyphyletic group, Adaptive evolution

## Abstract

**Background:**

The order Oedogoniales within the single family Oedogoniaceae comprised of three genera, *Oedogonium*, *Oedocladium,* and *Bulbochaete* based on traditional morphological criteria. While several molecular phylogenetic studies have suggested that both *Oedogonium* and *Oedocladium* may not be monophyletic, broader taxon sampling and large amounts of molecular data acquisition could help to resolve the phylogeny and evolutionary problems of this order. This study determined five chloroplast (cp) genomes of *Oedogonium* species and aimed to provide further information on cp genome for a better understanding of the phylogenetic and evolutionary relationships of the order Oedogoniales.

**Results:**

The five *Oedogonium* cp genomes showed typical quadripartite and circular structures, and were relatively conserved in their structure, gene synteny, and inverted repeats boundaries in general, except for small variation in genome sizes, AT contents, introns, and repeats. Phylogenetic analyses based on 54 cp protein-coding genes examined by maximum likelihood and Bayesian analyses using amino acid and nucleotide datasets indicated that both *Oedocladium* and *Oedogonium* are polyphyletic groups. A positively selected gene (*psb*A) was identified in the two *Oedocladium* species and the terrestrial *Oedogonium* species, indicating that terrestrial Oedogoniales taxa may have undergone adaptive evolution to adjust to the difference in light intensity between aquatic and terrestrial habitats.

**Conclusions:**

Our results enrich the data on cp genomes of the genus *Oedogonium*. The availability of these cp genomes can help in understanding the cp genome characteristics and resolve phylogenetic and evolutionary relationships of the order Oedogoniales.

**Supplementary Information:**

The online version contains supplementary material available at 10.1186/s12864-021-08006-1.

## Background

The order Oedogoniales within the single family Oedogoniaceae includes three genera: *Oedogonium* Link ex Hirn, *Oedocladium* Stahl, and *Bulbochaete* Agardh [1–4]. More than 600 species have been described in this order, most of which can be found in fresh waters throughout the world, although *Oedocladium* species are mainly found on soil surfaces, a few species of *Oedogonium* are found in moist soil surfaces [4–14]. The presence of branches and hairs are the genus-level characteristics to distinguish this order; *Oedogonium* has simple and unbranched filaments, *Bulbochaete* has bulb-based hairs, and *Oedocladium* has branched filaments [5–14]. While some molecular phylogenetic studies on Oedogoniales have suggested that both *Oedogonium* and *Oedocladium* do not appear to be monophyletic, and the morphological criteria of Oedogoniales do not define natural groups, making its evolutionary position unclear [15–20].

Chloroplast (cp) genomes have been found to be ideal for phylogenetic analysis and molecular evolution studies owing to advantages such as low evolution rate and maternal inheritance [21–25], and plastome has been increasingly used for phylogenetic and evolutionary studies of green algae. For example, Claude Lemieux et al. [26] conducted cp phylogenetic analysis based on the cp genes of 61 chlorophytes and revealed that Trebouxiophyceae is not monophyletic. Zhang et al. [27] demonstrated the adaptive mechanism of sea-ice environment by analyzing the molecular evolution of an Antarctic sea ice alga *Chlamydomonas* sp. based on cp protein-coding genes. However, only four cp genomes of Oedogoniales are currently available in public databases [28, 29], restricting the phylogenetic analysis and molecular evolution studies based on cp genomes of this group.

Nucleotide substitution rates are often used as the criterion to reflect selection pressure. While nonsynonymous substitution rates (dN) can cause amino acid change, synonymous substitution rates (dS) do not cause amino acid change. The dN/dS ratio is the measure of natural selection acting on the protein. According to Yang [30], dN/dS < 1 denotes negative purifying selection, dN/dS = 1 signifies neutral evolution, and dN/dS > 1 indicates positive selection [31]. As most of the plastid protein-coding genes undergo negative or purifying selection to maintain their function, they are conserved and have a low dN/dS ratio. However, some genes might undergo positive selection in response to environmental changes, consequently presenting relatively high dN/dS ratio [32–34].

In this study, the cp genomes of five *Oedogonium* species, were sequenced and an in-depth analysis of these genomes, including comparative analysis with previously reported *Oedocladium* and *Oedogonium* cp genomes, was performed. Furthermore, phylogenetic analysis and evolutionary study of the order Oedogoniales were conducted based on cp protein-coding genes and a positively selected gene was identified in *Oedocladium* species. The results of this study could be useful to understand the phylogenetic and evolutionary relationships of Oedogoniales.

## Results

### Species identification

**Fig. 1:**
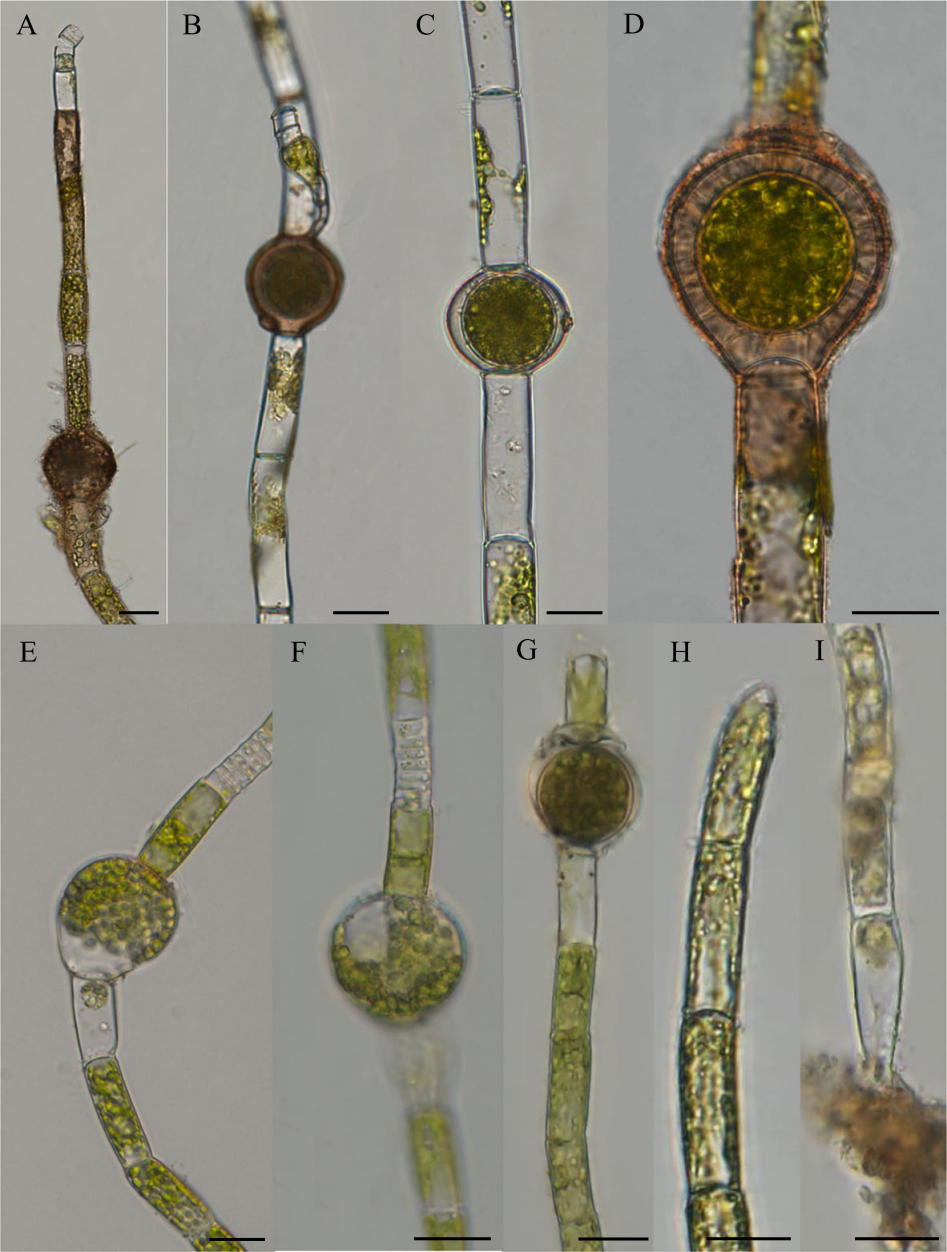
Photos of habitat and light microscopy of *Oe. dentireticulatum* and *Oe. crispum*. **A**-**D** *Oe. dentireticulatum*. **A** Showing the unbranched filament with oogonium, dwarf males and androsporangia. **B** Showing the dwarf male with two seriate and the oogonium. **C** Showing the median pore. **D** Sowing the oospore with dentate teeth. **E**-**I** *Oe. crispum.* **E**. Showing the unbranched filament with oogonium and antheridium. **F** Showing the sperms division horizontal, sperms 2. **G** Showing the oogonium single, obovoid-globose, operculate, division superior. **H** Terminal cell apically obtuse. **I** Elongated basal cell

**Fig. 2:**
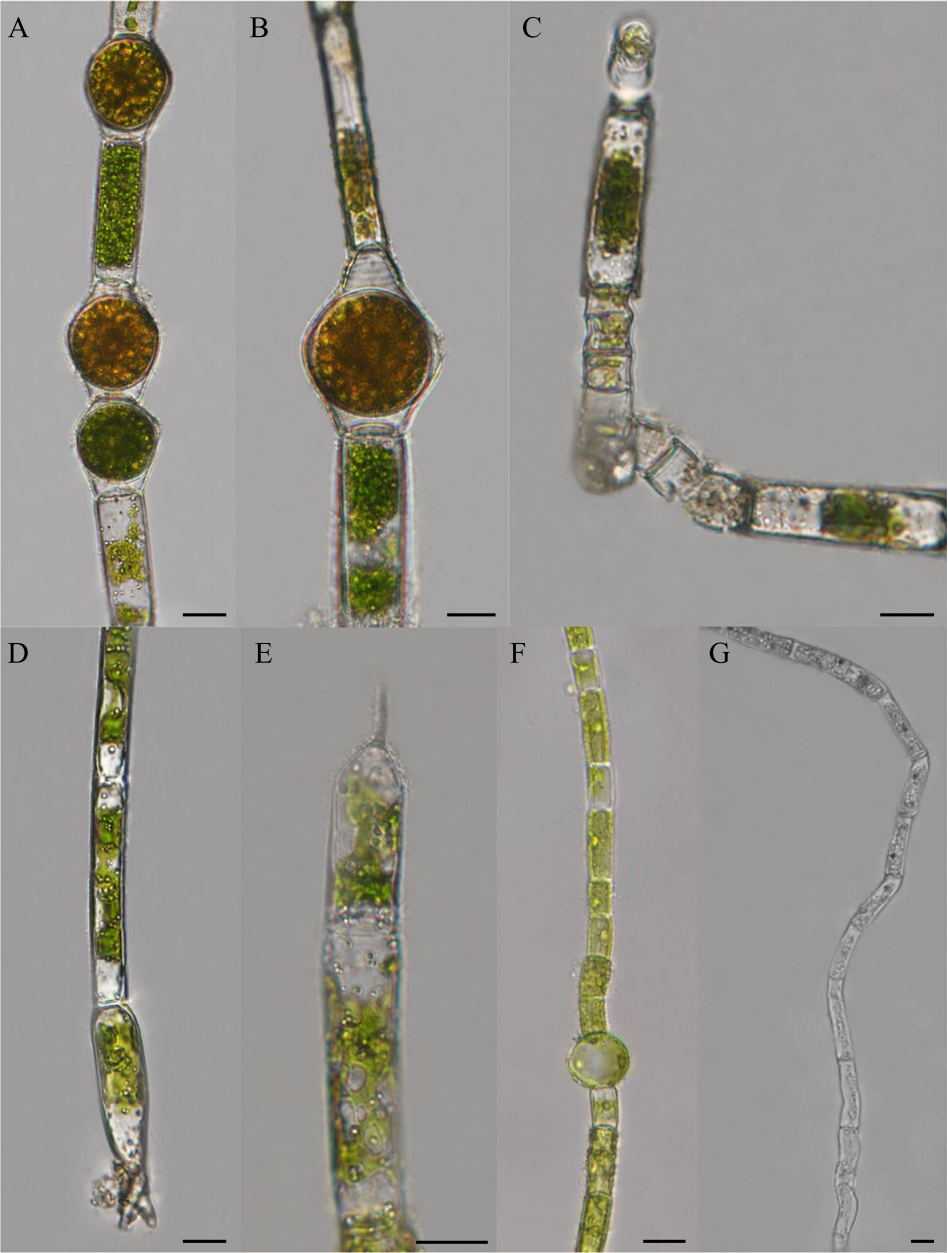
Photos of habitat and light microscopy of Oe. capilliforme and two undefined species. **A**-**E** *Oe. capilliforme*. **A** Unbranched female filament with oogonium single or 2-continues, oogonium with superior pore. **B** Oogonium with median pore. **C** Antheridium in 2–7 in a series, with sperms 2, division horizontal. **D** Apiculate terminal cell. **E** Elongated basal cell. **F** *Oe*. sp. (strain FACHB-3311), showing the unbranched filament with young oogonium. **G** *Oe*. sp. (strain FACHB-3313), unbranched filament with rhizoid (4% formaldehyde fixed sample). Scale bars: 20 μm

The characteristics of the five *Oedogonium* taxa were list in Table 1. And the sizes of these characters of each taxa were list in Supplementary. Light microscopy of the five *Oedogonium* taxa were in Figs. 1 and 2. For strain FACHB-3309 (Fig. 1A-D), the main features were almost the same with *Oe. dentireticulatum* Jao [9], except for the apical and basal cells were not observed, and both the samples showed the same locality in Chongqing Province in China, it was identified as *Oe. dentireticulatum* Jao morphologically. For strain FACHB-3310 (Fig. 1E–I), it was identified as *Oe. crispum* (Hassall) Wittrock for the length-width ration was different from the similar *Oe. autumnale* Wittrock with larger length-width ration, and it was also different from the similar *Oe. obesum* (Wittrock) Hirn with the oospore nearly or completely filling the oogonium instead of not filling the oogonium [9, 14]. Strain FACHB-3313 (Fig. 2A–E) was identified as *Oe. Capilliforme* Kuetzing, Wittrock [9, 14], it differed with the similar *Oe. plagiostomum* Wittrock not with the thickened spore wall. With regard to strains FACHB-3311 (Fig. 2F) and FACHB-3313 (Fig. 2G), the entire sexual features could not be observed; however, the filaments of both of these strains were unbranched, indicating that they obviously belonged to the genus *Oedogonium*. In particular, strain FACHB-3313 exhibited unbranched rhizoids that resembled those of *Oedocladium*.

**Table 1:** Matrix of phenotypic traits scored for the five Oedogoniales strains. Character state definitions are below, unknown character states are notated as “?”. Polymorphic conditions are indicated with multiple state numbers

Taxon (strain)	Characters and states													
	1	2	3	4	5	6	7	8	9	10	11	12	13	14
*Oe. dentireticulatum* (FACHB-3309)	0	2	0	0	1	0	0	1	1	1	1	?	?	0
*Oe. crispum* (FACHB-3310)	0	0	1	0	0	1	1	0	0	0	0	0	0	0
*Oe*. sp. (FACHB-3311)	0	?	?	?	?	?	?	?	?	?	?	?	?	0
*Oe. capilliforme* (FACHB-3312)	0	1	2	0	0	0	1	2	0	0	0	0	1	0
*Oe.* sp. (FACHB-3313)	0	?	?	?	?	?	?	?	?	?	?	?	?	0

1-Habit: unbranched filaments (0); branched filaments (1). 2-Sex differentiation: monoecious species (0); dioecious, macrandrous species (1); dioecious, nannandrous, gynandrosporous species (2); dioecious, nannandrous, idioandrosporous (3). 3-Oogonium’s morphology: subglobose (0); obovoid-globose (1); obovoid to subovoid (2). 4-If oospore filling the oogonium: same as oogonium, nearly or completely filling the oogonium (0). 5-Oospore wall ornamentation: smooth (0); outer layer reticulate and dentate, teeth spreading form reticulations (1). 6-Type of oogonial aperture: pore (0); circumcision (1). 7-Position of oogonial aperture: median to inframedian (0); superior (1). 8-If the antheridia single or continuous: single (0); 1 or 2 continuous (1); 2–7 in a series (2). 9-The number of sperm and the way of division: sperms 2, division horizontal (0); undefined (1). 10-Morphology of dwarf male: absent (0); on suffultory cells, stipes slightly curved, antheridia exteriors, 1 or 2 continuous (1); 11-Suffultory cells: uninflated (0); slightly inflated or inflated (1). 12-Basal cell: elongated (0); sub-hemispherical (1); undefined (2). 13-Terminal cell: obtuse (0); apiculate (1); 14-Vegetative cell’s morphology: cylindrical (0)

### General characteristics and comparison of Oedogoniales cp genomes

Table 2 summarizes the cp genomes characteristics of the five newly included *Oedogonium* species, three reported *Oedocladium* taxa and one *Oedogonium* species. The complete cp genomes of the nine species of Oedogoniales ranged from 146,367 bp (*Oe. crispum*) to 204,438 bp (*O. carolinianum*) in length. All of the five *Oedogonium* cp genomes displayed typical circular mapping with a large single copy (LSC) region (76,475–98,887 bp), a small single copy (SSC) region (43,305–58,055 bp), and two inverted repeats (IR) regions (12,808–35,492 bp) (Supplementary Figs. S1, S2, S3, S4, S5). The overall AT content in each cp genome was comparable and showed a little difference among the species, ranging from 69.98% (strain FACHB-3311) to 72.66% (*O. prescottii*); besides, difference was noted in coding proportion, which varied from 51.4% (*O. carolinianum*) to 69.5% (*O. prescottii*). The cp genomes of six *Oedogonium* species were moderately compact relative to those of the *Oedocladium* species. All the cp genomes contained 68 protein-coding genes and three rRNA genes, except for the cp genome of *Oe. cardiacum,* which have two additional genes (*dpo*B and *int*) located in the IR region. With respect to tRNA, the cp genomes showed slight difference as follows: *Oe. cardiacum* exhibited two additional *trn*R(ccu) located in the IR regions and *Oe*. *dentireticulatum* (strain FACHB-3309) presen; ted an additional *trn*R(ccu) in the LSC region; both *Oe*. *crispum* and *Oe*. sp. (strain FACHB-3313) contained two additional *trn*R(ccg) in the IR regions and *O. carolinianum* has an additional *trn*R(ccg) in the LSC region; and *O. carolinianum* has an additional *trn*S(gga) in the LSC region. Sequence repeats of more than 30 bp were less frequent (3.9–4.9%) in the cp genomes of the five *Oedogonium* species when compared with those in the two *O. carolinianum* cp genomes, but were more frequent, when compared with those in the *Oe*. *cardiacum* cp genome.

**Table 2:** General features of nine oedogonialean chloroplast genomes

Genomic Feature	*O. prescottii*	*O. carolinianum* (FACHB-2453)	*O. carolinianum*	*Oe. cardiacum*	*Oe. dentireticulatum*	*Oe. crispum*	*Oe.*sp. (FACHB-3311)	*Oe. capilliforme*	*Oe.*sp. (FACHB-3313)
Size (bp)									
Total	154,9 78	200,832	204,438	196,547	159,341	146,367	187,104	195,349	179,946
IR	12,808	22,275	23,748	35,492	19,159	13,284	25,841	35,138	26,999
LSC	80,821	98,462	98,887	80,363	77,718	76,475	86,403	80,003	80,347
SSC	48,542	57,820	58,055	45,200	43,305	43,324	49,019	45,070	45,601
A + T(%)	72.66	70.51	70.2	70.5	71.46	72.28	69.98	70.64	71.23
Coding proportion^a^	67.0%	52.7%	51.4%	55.9%	64.1%	69.5%	54.8%	52.8%	55.9%
Gene (+/−)^bc^	49/60	50/61	50/61	52/63	50/59	50/61	49/60	49/60	50/61
Protein-coding genes(number/proportion)	68 29/43	68 28/44	68 28/44	70 31/45	68 29/43	68 29/43	68 29/43	68 29/43	68 29/43
rRNA(number/proportion)	3	3	3	3	3/3	3/3	3/3	3/3	3/3
tRNA(number/proportion)	28 17/14	30 19/14	30 19/14	29 18/15	29 18/14	29 18/15	28 17/14	28 17/14	29 18/15
Introns	6	15	17	21	22	8	37	24	30
Group I (no.)	1	5	7	17	18	4	26	20	25
Group II (no.)	5	10	10	4	4	4	11	4	5
Repeats^d^(%)	4.8	8.9	11.3	1.3	3.9	4.0	4.7	3.3	4.9
Accession number	MT364368	MT364369	NC_031510	EU677193	MW250871	MW250872	MW250873	MW250874	MW250875

^a^The coding proportion only includes all annotated protein-, rRNA-, and tRNA-coding regions; ^b^Gene and CDS numbers do not include ORF genes; ^c^The plus-minus sign means number of genes in plus strain (left side of slash) or minus strain (rightside of slash). ^d^Non-overlapping repeat elements were mapped on each genome with RepeatMasker using as input sequences the repeats ≥30 bp identified with Vmatch

### Introns content and insertion sites

The introns content and insertion sites of the nine Oedogoniales cp genomes are listed in Table 2 and Supplementary Tables S1 and S2. The nine cp genomes significantly differed with respect to the introns content. *Oe*. sp. (strain FACHB-3311) has the maximum introns content with 26 group I introns and 11 group II introns. When compared with the other six *Oedogonium* cp genomes, multiple intron losses were observed in the cp genome of *Oe*. *crispum* (strain FACHB-3310), with four group I introns in *trn*L(uaa), *psb*C, *atp*A, and *psb*D, respectively, and four group II introns in *psb*I, *pet*D, *psa*C, and *psa*B, respectively. Besides, similar to *O. prescottii*, *Oe*. *crispum* also exhibited introns losses in *psb*A. *Oe*. sp. (strain FACHB-3311) presented two additional group II introns in *chl*B and *chl*L, introns were first observed in them. All the nine cp genomes included group I introns in *trn*L(uaa), which is common across all algal lineages and is considered to originate from the common ancestor of cp [35]. The nine cp genomes showed a certain variation in insertion sites. The common group I introns in *trn*L(uaa) and group II introns in *pet*B, *psa*C, and *psb*I (only strain FACHB-3311 lost the intron in *psb*I) showed the same insertion sites. With regard to the other genes with introns, the insertion sites in different species showed similarities and variations. For instance, in *psb*A, the number of introns (introns in *psb*A are all group I) differed among the species, whereas the insertion sites of the first intron in *Oe*. *dentireticulatum* (strain FACHB-3309), *Oe*. sp. (strain FACHB-3311), and *Oe*. sp. (strain FACHB-3313) were identical. The two *O. carolinianum* were the same; however, the insertion site of the first intron in *Oe. capilliforme* was similar to that of the fourth intron in *Oe. dentireticulatum* and sp. (strain FACHB-3311).

### Synteny analysis and average nucleotide identity analysis

**Fig. 3:**
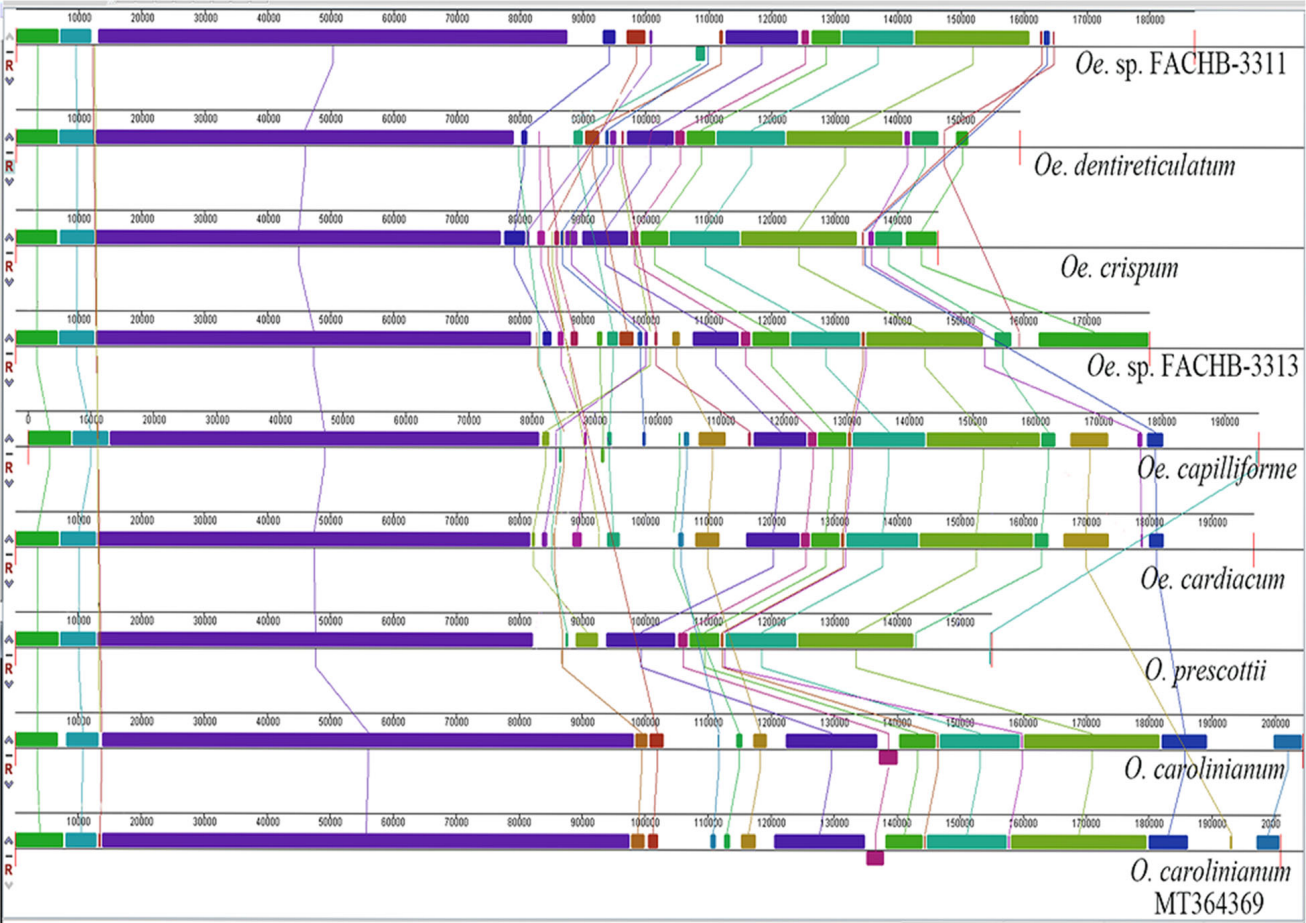
Synteny comparison of Oedogoniales algae chloroplast genomes using progressiveMauve. The coloured syntenic blocks are local collinear blocks; blocks above the centre line indicate they are on the same strand, and blocks below the center line indicate they are on the opposite strand

ProgressiveMauve was used to analyze the Oedogoniales cp genomes synteny, with *Oe. cardiacum* used as a reference to compare gene order among the cp genomes (Fig. 3). More than 19 locally collinear blocks (LCBs) were identified in the cp genomes of the nine species of Oedogoniales, including six taxa from *Oedogonium* and three taxa from *Oedocladium*. The nine cp genomes showed high degree of syntenic conservation on the whole, with *Oe. capilliforme* exhibiting high similarity to *Oe. cardiacum*, and *Oe. dentireticulatum* resembling *Oe*. sp. (strain FACHB-3311). However, some rearrangements and inversions were still observed among certain short LCBs mainly owing to the inversion or loss of introns. The genes order and number were almost identical except for that an inversion between *trn*E(uuc) and *pet*L with a length of less than 3 kb and including the genes *pet*D and *trn*R(ucg) was detected in *O. carolinianum* (MT364369) and *O. carolinianum* (NC_031510).

The average nucleotide identity (ANI) of the nine species of Oedogoniales was calculated using FastANI (Supplementary Fig. S6). *Oe. crispum* showed high ANI with *Oe. dentireticulatum* and *Oe*. sp. (strain FACHB-3311) (90.64 and 90.56%, respectively), *Oe. dentireticulatum* was similar to *Oe*. sp. (strain FACHB-3311) with 92.57% ANI, and *Oe. capilliforme* was similar to *Oe. cardiacum* with 97.03% ANI.

### IR expansion and contraction

**Fig. 4:**
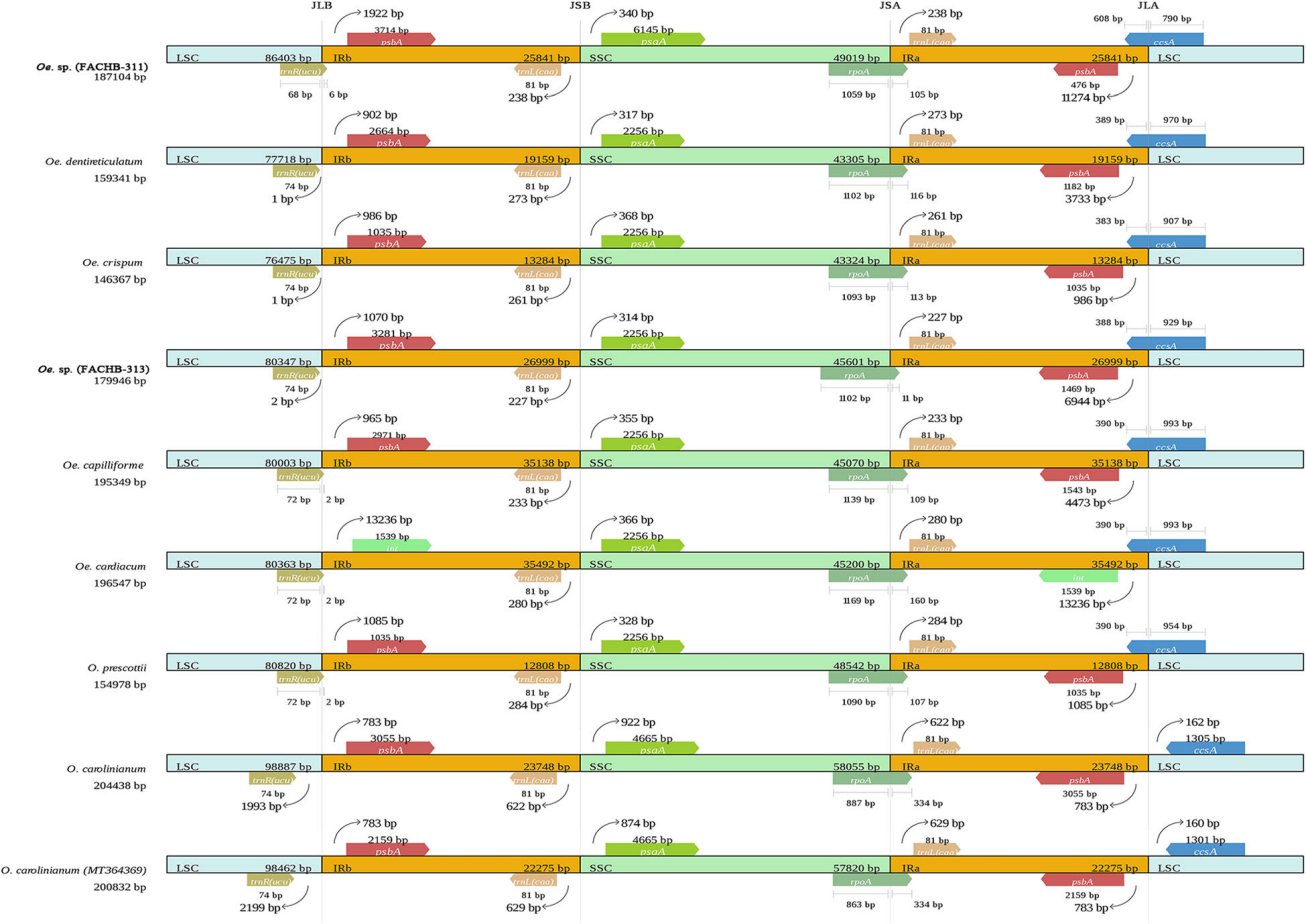
Comparison of the IR-SC boundaries among nine Oedogoniales species

The IR boundary regions of the nine species of Oedogoniales were compared as illustrated in Fig. 4. *Oe. cardiacum* and *Oe. capilliforme* (strain FACHB-3312) showed larger IRs reaching 35,000 bp, whereas *Oe. crispum* and *O. prescottii* exhibited smaller IRs reaching 13,284 and 12,808 bp, respectively. The IRs of all the nine cp genomes contained the same four protein-coding genes, three tRNAs, and three rRNAs. However, in *Oe. crispum* and *Oe*. sp. (strain FACHB-3313), an additional *trn*R(ccg) was observed between *psb*A and *rbc*L; *Oe. cardiacum* included two additional protein-coding genes (*int* and *dpo*B) and one tRNA (*trn*R(ccu)); and the IRa of four cp genomes included parts of the 5′-end of *ccs*A (390 bp in *Oe. cardiacum*, *Oe. capilliforme,* and *O. prescottii* and 383 bp in *Oe. crispum*).

The nine Oedogoniales cp genomes showed high conservation at four regional boundaries, with little variation. The LSC/IRb junctions (JLBs) in the cp genomes of *Oe. cardiacum*, *Oe. capilliforme*, *O. prescottii,* and *Oe*. sp. (strain FACHB-3311) were located in *trn*R(ucu); as a result, 2 bp of the 3′-end of this gene were a part of the IR region. In *Oe*. sp. (strain FACHB-3311), the IR region contained 6 bp of the 3′-end of *trn*R(ucu), and in the other five cp genomes, the LSC/IRb boundaries occurred between *trn*R(ccu) and *psb*A. The IRb/SSC boundaries in all the nine cp genomes occurred between *trn*L(caa) and *psa*A, and the SSC/IRa junctions were located in *rpo*A. The IRa/LSC junctions (JSAs) of the two *O. carolinianum* cp genomes occurred between *psb*A and *ccs*A, while those of the other seven genomes were located in *ccs*A, with 390 bp of the 5′-end of this gene being a part of the IR region in *Oe. cardiacum*, *Oe. capilliforme,* and *O. prescottii*, and 383, 388, 608, and 389 bp of the 5′-end of this gene being a part of the IR region in *Oe. crispum*, strain FACHB-3313, strain FACHB-3311, and *Oe. dentireticulatum,* respectively.

### Phylogenetic analysis and adaptive evolution analysis

**Fig. 5:**
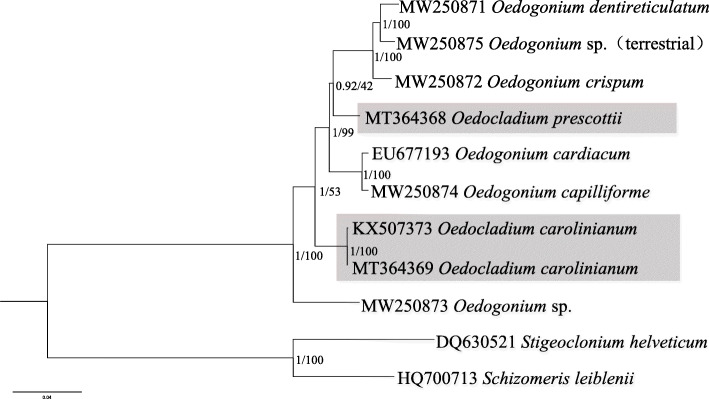
Phylogenetic tree based on 54 chloroplast genes was generated by the amino acid data sets. Numbers on the left and right side at the branches represent Bayesian posterior probabilities and bootstrap values, respectively. Scale bar indicates substitutions per site

**Fig. 6:**
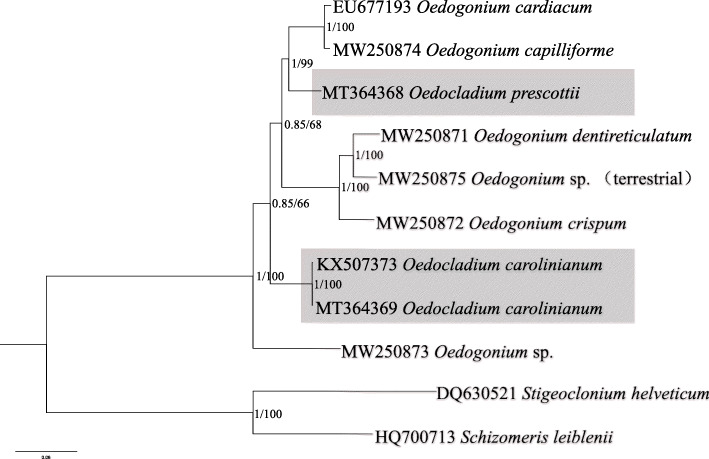
Phylogenetic tree based on 54 chloroplast genes was generated by the nucleotide data sets. Numbers on the left and right side at the branches represent Bayesian posterior probabilities and bootstrap values, respectively. Scale bar indicates substitutions per site

Phylogenetic assays based on 54 cp protein-coding genes were conducted using maximum likelihood (ML) and Bayesian analyses with amino acid and nucleotide datasets, which generated two kinds of phylogenetic trees showing the same results (Figs. 5 and 6). Phylogenetic trees based on amino acid and nucleotide datasets both indicated that the nine species of Oedogoniales clustered into three clades *Oe*. sp. (MW250873) formed a separate clade with absolute high support value, the two *O. carolinianum* clustered together and formed another clade, and the other six Oedogoniales formed the third clade. With regard to the third clade, the two datasets showed a little difference in the location of *O*. *prescottii*. Based on nucleotide dataset, *O*. *prescottii* clustered with *Oe*. *cardiacum* and *Oe*. *capilliforme*, whereas according to the amino acid dataset, *O*. *prescottii* clustered with *Oe*. *dentireticulatum*, *Oedogonium* sp. (MW250875), and *Oe*. *crispum*. A total of 26 taxa, including the newly added five *Oedogonium* taxa, were included in the 18S rDNA phylogeny (Supplementary Fig. S7). The phylogenetic tree showed that species of *Bulbochaete* was separated with *Oedogonium* and *Oedocladium* with absolutely high support value, and the species of *Oedocladium* formed two branches separated by two species of *Oedogonium*, the five newly included *Oedogonium* species separated with each other distributed in the other small clades. All these results indicated that both *Oedocladium* and *Oedogonium* are polyphyletic, which is in accordance with that reported in a previous study [36].

Based on the ML method of 54 chloroplast protein-coding genes, the value of dN and dS were compared between terrestrial and aquatic species of Oedogoniales. (Supplementary Table S3). No genes showed significantly different between the two group of algae at the levels of dN and dS. The ML method is a pairwise approach to estimate the dN/dS ratio, a dN/dS ratio may indicate in one or both species, and some specific sites under positive selection may remain undetected [37]. Positive selection analysis was performed based on branch-site model, and the null and alternative models were compared. The null model considered that the foreground branch only has dN/dS = 1, and the alternative model assumed that sites on the foreground branch have dN/dS > 1 (positive selection). When the two *Oedocladium* species and MW250875 were labelled as the foreground branch, the FDR-adjusted *P* value of *psb*A was less than 0.05 (Table 3). Based on Bayes empirical Bayes (BEB) assay, it indicated that *psb*A may possibly contain sites under positive selection, with 291SER showing posterior probability higher than 99%. However, owing to the lack of related functional sites information on closely related species such as *Chlamydomonas reinhardtii* and *Stigeoclonium helveticum* in UniProt, the positively selected sites of *psb*A require further investigation.

**Table 3:** Positively selected sites in terrestrial Oedogoniales species (*Oedocladium* species and *Oedogonium* sp. (MW250875))

Gene	lnL H0	lnL HA	df	lnL 2 × |(HA-H0)|	P value	FDR	Positive selected sites under BEB analysis
*psb*A	− 2309.7534	− 2299.2283	1	21.0502	0.0000	0.0002	**291S-0.998**299G-0.920

Number behind hyphen is the posterior probability of BEB analysis

## Discussion

In this study, we investigated five *Oedogonium* isolates from China, of which strains FACHB-3309, FACHB-3310, and FACHB-3312 were identified as *Oe*. *dentireticulatum*, *Oe*. *crispum*, and *Oe*. *capilliforme*, respectively. Strains FACHB-3311 and FACHB-3313 were considered to belong to the genus *Oedogonium* owing to their unbranched filaments; however, they could not be identified at species level owing to their lack of entire sexual characters.

Comparative analyses of the nine Oedogoniales cp genomes showed highly conserved structures and gene numbers. The cp genomes of the newly sequenced five *Oedogonium* species were found to share the same structure as the previously reported Oedogoniales cp genomes, and the structures of the tetrad were not altered, but were different from the other two orders in the OCC clade (the IR is obliterated in the reported cp genomes in Chaetophorales and *Floydiella* of Chaetopeltidales). It has been indicated IR loss may be a synapomorphy marking the common ancestry of Chaetophorales and Chaetopeltidales [38]. The total length of these cp genomes was observed to vary within a relatively large range, extending from 146,367 bp (*Oe*. *crispum*) to 204,438 bp (*O. carolinianum*), which may be the result of contraction and expansion of IR regions and proportion of non-coding sequences, such as the introns content. Furthermore, the nine cp genomes showed highly conserved protein-coding genes and rRNAs number; however, they presented a slight difference in the tRNAs content. With regard to the introns content, the nine cp genomes exhibited relative variation, and the number of group I introns significantly differed, mainly owing to the diversity in the introns in *psb*A. In particular, introns (group II) were observed for the first time in *chl*B and *chl*L in *Oe*. *dentireticulatum*. All the nine cp genomes retained the group I introns in *trn*L(uaa) and group II introns in *pet*D and *psa*C, and shared the same insertion sites. With regard to the other genes with introns, the insertion sites of different species showed similarities and variations.

Synteny analyses revealed a relatively high degree of syntenic conservation among the nine cp genomes, and only one inversed segment was detected in *O. carolinianum* FACHB-2453 and *O. carolinianum* UTEX LB 1686. The other variations were mainly owing to the introns, and no structural variation was observed in the six *Oedogonium* species. The results of FastANI also supported the findings of synteny analyses, indicating that *Oe. capilliforme* had high similarity with *Oe. cardiacum*, and *Oe. dentireticulatum* resembled strain FACHB-3311.

IR regions are the most conserved regions in the cp genomes. Frequent expansions and contractions at the junctions of SSC and LSC with IRs illustrate the relationships among the taxa and have been recognized as evolutionary signals [39–43]. The nine species of Oedogoniales examined in the present study showed only a few variations at the junctions. When compared with the two *O. carolinianum*, *O. prescottii* showed higher similarities to the five *Oedogonium* species, and the five *Oedogonium* species were similar to each other. The IR regions of *O. prescottii* and *Oe*. *crispum* presented a contraction, when compared with those of the other Oedogoniales taxa, and the cp genomes of both *O. prescottii* and *Oe*. *crispum* exhibited the shortest length. Previous studies have indicated that IR expansion and contraction frequently result in variations in genome size, which can be applied to phylogenetics and genome evolution analyses [40, 41, 44], and gene conversion during speciation is considered to be responsible for small IR expansions or contractions [40, 41, 45–47].

Phylogenetic studies based on 54 cp protein-coding genes assayed using ML and Bayesian analyses with amino acid and nucleotide datasets and 18S rDNA all showed that *Oedocladium* and *Oedogonium* are polyphyletic, which is in accordance with that reported previously. However, the support value based on nucleotide dataset and 18S rDNA was not very high at the basal node, probably owing to the lack of sufficient representative taxa for this group as well as different evolutionary rates of the amino acid sequence and nucleotide data. Previous studies have proposed that larger sample sizes can substantially improve the phylogenetic results [48].

Positively selected genes are known to play a key role in adaptation to different environments and speciation [49–53], and it is necessary to understand the adaptive evolutionary history of *Oedocladium* species. The results of the present study showed that 291SER of *psb*A may be under positive selection with posterior probability higher than 99%. The genus *Oedocladium* (terrestrial) is presumed to have partly originated from *Oedogonium* species, which grow on moist soil surface and present underground filaments with slightly unbranched rhizoids [9]. The *psb*A encodes the photosystem II reaction center protein D1, which is one of the two reaction center proteins of photosystem II. Photosystem II is the first link in the chain of photosynthesis, and captures photons and uses the energy to extract electrons from water molecules [54].It has been reported that the genes in the cp genome (including *psb*A) of *Curcuma* sp. show adaptive evolution to adapt to the changes in light conditions [55], and that the green alga *Chlamydomonas* sp. ICE-L underwent adaptive evolution to adapt to extreme polar environment [27]. We speculate that the *Oedocladium* species and terrestrial *Oedogonium* species could have partly originated from the aquatic *Oedogonium* species, and might have undergone adaptive evolution during this process to adapt to the difference in light intensity between aquatic and terrestrial habitats. Nevertheless, more genomic data, especially for terrestrial species, may help to verify these hypotheses and further understand the phylogenetic and evolutionary relationships of the order Oedogoniales.

## Conclusion

The present study determined the cp genomes of five *Oedogonium* speciesand revealed that the overall structure and gene contents of the Oedogoniales cp genomes were relatively conserved, except for some variations in genome sizes, AT contents, introns, and repeats. Phylogenetic analysis based on 54 cp protein-coding genes and 18S rDNA genes all indicated that both *Oedogonium* and *Oedocladium* are polyphyletic. The positively selected gene in the two *Oedocladium* species was identified, and the terrestrial *Oedogonium* species were speculated to have undergone adaptive evolution to adapt to the difference in light intensity between aquatic and terrestrial habitats. These findings not only strengthen our understanding of Oedogoniales cp genomes, but also help us to comprehend the phylogenetic and evolutionary relationships of the order Oedogoniales.

## Methods

### Sampling, culture conditions, DNA extraction, and morphological observation

The strains described in this study were isolated from water or soil samples, and have been deposited to the Freshwater Algae Culture Collection at the Institute of Hydrobiology (FACHB collection), Wuhan, Hubei Province, China. Strain FACHB-3309 was collected from a paddy field in Hechuan (29°50′15″ N, 106°12′46″ E), Chongqing Province, China, in March 2019. Strain FACHB-3310 was collected from a pond in Lvliang (37°34′20″ N, 112°12′29.25″ E), Shanxi Province, China, in July 2018. Strain FACHB-3311 was collected from a pond in Wuhan (30°3′46″ N, 114°23′56″ E), Hubei Province, China, in June 2019. Strain FACHB-3312 was collected from a ditch in Wuhan (30°33′2″ N, 114°25′48″ E), Hubei Province, China, in April 2019. Strain FACHB-3313 was collected from damp soil in a park in Haikou (20°2′23″ N, 110°21′1″ E), Hainan Province, China, in December 2018. All the strains were grown at 25 °C in liquid BG11 medium under a 12/12-h light/dark cycle. The genomic DNA was extracted using a Universal DNA Isolation Kit (Axygen, Suzhou, China) [56]. An Olympus BX53 (Tokyo, Japan) light microscope equipped with an Olympus DP80 digital camera and CellSens standard image analysis software (Tokyo, Japan) were used for morphological examination. The characteristics of the five species were summarized in Table 1.

### Library preparation, sequencing, genome assembly, and annotation

A NEB Next Ultra DNA Library Prep Kit for Illumina (New England Biolabs, Ipswich, MA, USA) was used for preparing sequencing libraries, which were sequenced on an Illumina NovaSeq 6000 platform by a commercial provider (Novogene, Beijing, China). The methods of genome assembly and annotation have been described elsewhere [36, 57]. The raw data were trimmed using SOAPnuke software [58] to remove the low-quality and the adapter sequences (the reads of the five species were with a mean length about 150 bp) and then assembled using SPAdes [59]. The resulting assembly contigs were considered to have originated from the cp genome if the (1) BLAST searches in publicly available cp genomes returned Chlorophyta species with significant e-values (1e-5); (2) GC content of the contigs was less than 45% (the GC content of previously sequenced green algal cp genomes is typically less than 45%); and (3) sequencing depth was more than 100-fold coverage. Subsequently, trimmed clean reads were aligned to the resulting assembly contigs using BWA-MEM [60]. If the reads mapped to two contigs, the order of the contigs was determined and one sequence was produced, which was confirmed by Sanger dideoxy sequencing. The cp genomes were initially annotated using GeSeq [61] with the reported Oedogoniales cp genomes as references. Protein-coding and ribosomal RNA genes were further polished using Blast with genes from the available Oedogoniales cp genomes. The tRNA genes were identified using tRNAscan-SE [62]. BLAST was used to refine the annotation results. Intron boundaries were determined by comparing intron-containing genes with homologs without introns, and intron subgroup affiliation was determined by modelling intron secondary structures [63, 64] using RNAweasel tool [65]. Forward and palindromic repeats larger than 30 bp were searched using Vmatch software (http://www.vmatch.de/) with the options -f -p -l -h -allmax and masked in the genome sequence by RepeatMasker (http://repeatmasker.org) running under the NCBI/RMBLAST (2.9.0+) search engine (http://blast.advbiocomp.com). The annotated sequences have been deposited to the NCBI GenBank database under the accession numbers MW250871–MW250875 (corresponding to strains FACHB-3309–FACHB-3313, respectively). Genome maps were generated using OrganellarGenomeDRAW [66].

### Phylogenetic analysis

Phylogenetic analysis of the algal strains was performed by examining the sequences of cp protein-coding genes based on amino acid and nucleotide datasets and the 18S rDNA. The amino acid and nucleotide datasets of the cp genomes were concatenated using the following 54 protein-coding genes: *atp*A, *atp*B, *atp*E, *atp*F, *atp*H, *atp*I, *cem*A, *chl*B, *chl*L, *chl*N, *clp*P, *pet*B, *pet*D, *pet*G, *pet*L, *psa*A, *psa*B, *psa*C, *psa*J, *psb*A, *psb*B, *psb*D, *psb*E, *psb*F, *psb*H, *psb*I, *psb*J, *psb*K, *psb*L, *psb*M, *psb*N, *psb*T, *psb*Z, *rpl*14, *rpl*16, *rpl*2, *rpl*20, *rpl*23, *rpl*36, *rpl*5, *rps*11, *rps*12, *rps*14, *rps*18, *rps*19, *rps*2, *rps*3, *rps*7, *rps*8, *rps*9, *tuf*A, *ycf*12, *ycf*3, *ycf*4. The amino acid sequences were aligned using MAFFT 7.0 [67], and those employed in the nucleotide dataset were additionally aligned using the MUSCLE function of MEGA7 [68] with the option “align codons” [69]. Ambiguous regions were removed from each alignment using trimAl 1.2 [70] with the option gt = 1. Evolutionary models and partitions of the datasets were determined using PartitionFinder 2 [71], and the best partitions are shown in Table 3. ML and Bayesian analyses were used for inferring phylogenies. IQ-TREE web server [72] was employed to perform ML analysis with 1000 ultrafast bootstraps [73] and 1000 SH-aLRT tests [73, 74] to examine nodal support. Bayesian analysis was conducted using MrBayesv3.2.6 [75], and the dataset was partitioned as shown in Table 4. Markov chain Monte Carlo analyses were run with four Markov chains (three heated, one cold) for 1,000,000 generations, and trees were sampled every 1000 generations. In each round of calculation, a fixed number of samples (burn-in = 1000) was discarded from the beginning of the chain. Reference sequences were downloaded from GenBank. 18S rDNA sequences were aligned using MAFFT 7.0 [67], and ambiguous regions were manually edited and adjusted by eye using MEGA7 [68]. Bayesian inference (BI) of the software program MrBayes v3.2.6 [75] was used, and an evolutionary model was determined using jModelTest2 with the best model was GTR + I + G [76]. An alignment of the cp genome sequences of all the species of Oedogoniales was generated using Mauve ver. 2.3.1 with the progressive mode [77]. FastANI [78] was employed to determine the ANI of all the cp genomes.

**Table 4:** Partition scheme of 54 concatenated chloroplast protein-coding genes used in this study

Subset	Amino acid data sets		Nucleotide data sets	
	Best model	Partition scheme	Best model	Partition scheme
1	LG + I + G	*rps*12*, chl*L*, atp*B*, atp*A	GTR + I + G	*atp*A*, rps*19*, tuf*A*, atp*F, *atp*I, *ycf*12*, pet*L
2	LG + G	*atp*F*, chl*N*, atp*E	GTR + I + G	*psa*J*, psb*Z*, psb*B*, psa*B*, pet*D*, psb*D, *psb*K*, pet*B*, ycf*3*, psb*H*, rpl*16*, atp*B*, rpl*14
3	MTZOA+G + F	*psb*M*, pet*B*, psb*I*, psb*E*, psb*T*, psb*N*, atp*H*, psb*D*, psb*B*, psa*A*, psa*B	GTR + I + G	*ycf*4*, rps*12*, rpl*2*, atp*E
4	CPREV+G	*rps*19*, atp*I*, chl*B*, rpl*16*, rpl*2*, rpl*5	GTR + G	*atp*H*, psb*A
5	CPREV+G	*rps*14*, rpl*20*, rps*9*, cem*A*, ycf*4	GTR + G	*psb*M*, psb*I*, psa*C*, psb*L*, psb*F*, psb*E*, cem*A*, rpl*36*, rps*14
6	JTT + G + F	*rps*18*, rps*8*, rpl*23*, rps*3*, clp*P	GTR + I + G	*psb*N*, chl*B*, chl*L*, rps*11*, rpl*5*, chl*N
7	MTZOA+G	*psa*J*, psb*F*, psb*J*, psb*K*, psb*H*, pet*L*, psb*Z*, pet*D	GTR + G	*clp*P*, rps*7
8	LG	*pet*G*, ycf*3	GTR + G	*psb*J*, psb*T*, pet*G
9	PMB + G	*rpl*36*, psb*A*, psa*C	GTR + I + G	*psa*A
10	LG	*rps*11*, psb*L*, rpl*14	GTR + G	*rps*2, *rps*8, *rps*9, *rpl*20, *rpl*23, *rps*18
11	LG + G	*rps*2	GTR + G	*rps*3
12	FLU+G	*rps*7		
13	LG4M + G	*tuf*A		
14	MTZOA	*ycf*12		

### Evolutionary analysis

The CODEML program of PAML v4.9 [30] with the ML model (runmode = − 2, CodonFreq = 2) was used to measure the values of dS and dN, the analysis was based on 54 chloroplast protein-coding genes. Comparisons of the evolutionary rates were conducted using the two-tailed Wilcoxon rank sum test. The multiple testing was corrected by applying the false discovery rate method (FDR) [79].Branch-site model was utilized to find genes that possibly underwent positive selection. The improved branch-site model (model = 2, Nsites = 2) was used to detect signatures of positive selection on individual codons in a specific branch [80]. The three *Oedocladium* species and the terrestrial *Oedogonium* sp. (strain FACHB-3313) were set as the foreground branch. The null model assumed that no positive selection occurred on the foreground branch (fix_omega = 1, omega = 1), and the alternative model assumed that sites on the foreground branch were under positive selection (fix_omega = 0, omega = 2). LRT were used to test model fit and Chi-square test was applied for examining the *P* values. A correction was performed for multiple testing using an FDR criterion, and BEB method was employed to statistically identify sites under positive selection. Genes with an FDR-adjusted *P* < 0.05 were considered as putatively selected.

## Supplementary Information

**Additional file 1: Supplementary Fig. S1**. Gene map of *Oe*. *dentireticulatum* chloroplast genomes.

**Additional file 2: Supplementary Fig. S2**. Gene map of *Oe*. *crispum* chloroplast genomes.

**Additional file 3: Supplementary Fig. S3**. Gene map of *Oe*. sp. (FACHB-3311) chloroplast genomes.

**Additional file 4: Supplementary Fig. S4**. Gene map of *Oe*. *capilliforme* chloroplast genomes.

**Additional file 5: Supplementary Fig. S5**. Gene map of *Oe*. sp. (FACHB-3313) chloroplast genomes. Arrows show the direction of transcription. The same colour block shows the functional gene group (legend at bottom left). Transfer RNAs are represented by their one-letter amino acid code. The grey circle on the inside shows a graph of the GC content.

**Additional file 6: Supplementary Fig. S6**. Heat map of ANI values of the nine Oedogoniales cp genomes.

**Additional file 7: Supplementary Fig. S7**. Phylogenetic tree of the Oedogoniales algae based on 18S rDNA sequences. Numbers at the branches represent Bayesian posterior probabilities (≥0.5). Branch lengths are proportional to the genetic distances, which are indicated by the scale bar (Bold species are the newly included in this study).

**Additional file 8: Supplementary Table S1**. Insertion sites of group I introns of the nine Oedogoniaes cp genomes [81].

**Additional file 9: Supplementary Table S2**. Insertion sites of group II introns of the nine Oedogoniaes cp genomes [81].

**Additional file 10: Supplementary Table S3**. Substitution rates in the chloroplast protein coding genes of Oedogoniales species.

## Data Availability

All data generated and analysed during this study are included in this published article and its supplementary information files. Raw sequencing data of all species are available from the National Center for Biotechnology Information (NCBI) (https://www.ncbi.nlm.nih.gov/). Accession numbers of cpDNA: MW250871-MW250875. MW250871: https://www.ncbi.nlm.nih.gov/nuccore/MW250871 MW250872: https://www.ncbi.nlm.nih.gov/nuccore/MW250872 MW250873: https://www.ncbi.nlm.nih.gov/nuccore/MW250873 MW250874: https://www.ncbi.nlm.nih.gov/nuccore/MW250874 MW250875: https://www.ncbi.nlm.nih.gov/nuccore/MW250875

## Abbreviations

OCC clade: Oedogoniales, chaetophorales, and chaetopeltidales
IR: Inverted repeats
ML: Maximum likelihood
cpDNA: Chloroplast DNA
Oe.: *Oedogonium*
O.: *Oedocladium*
FACHB: Freshwater Algae Culture Collection at the Institute of Hydrobiology
μm: Micrometer
BEB: Bayes empirical Bayes
