# Supplementary material for: Chloroplast genomes of five Oedogonium species: genome structure, phylogenetic analysis and adaptive evolution
Source: BMC Genomics. 2021 Sep 30;22:707. doi: 10.1186/s12864-021-08006-1 (PMC8485540; doi:10.1186/s12864-021-08006-1)
Supplement: Supplementary file 2 — Additional file 2: Supplementary Fig. S2. Gene map of Oe. crispum chloroplast genomes. [file 12864_2021_8006_MOESM2_ESM.pdf]

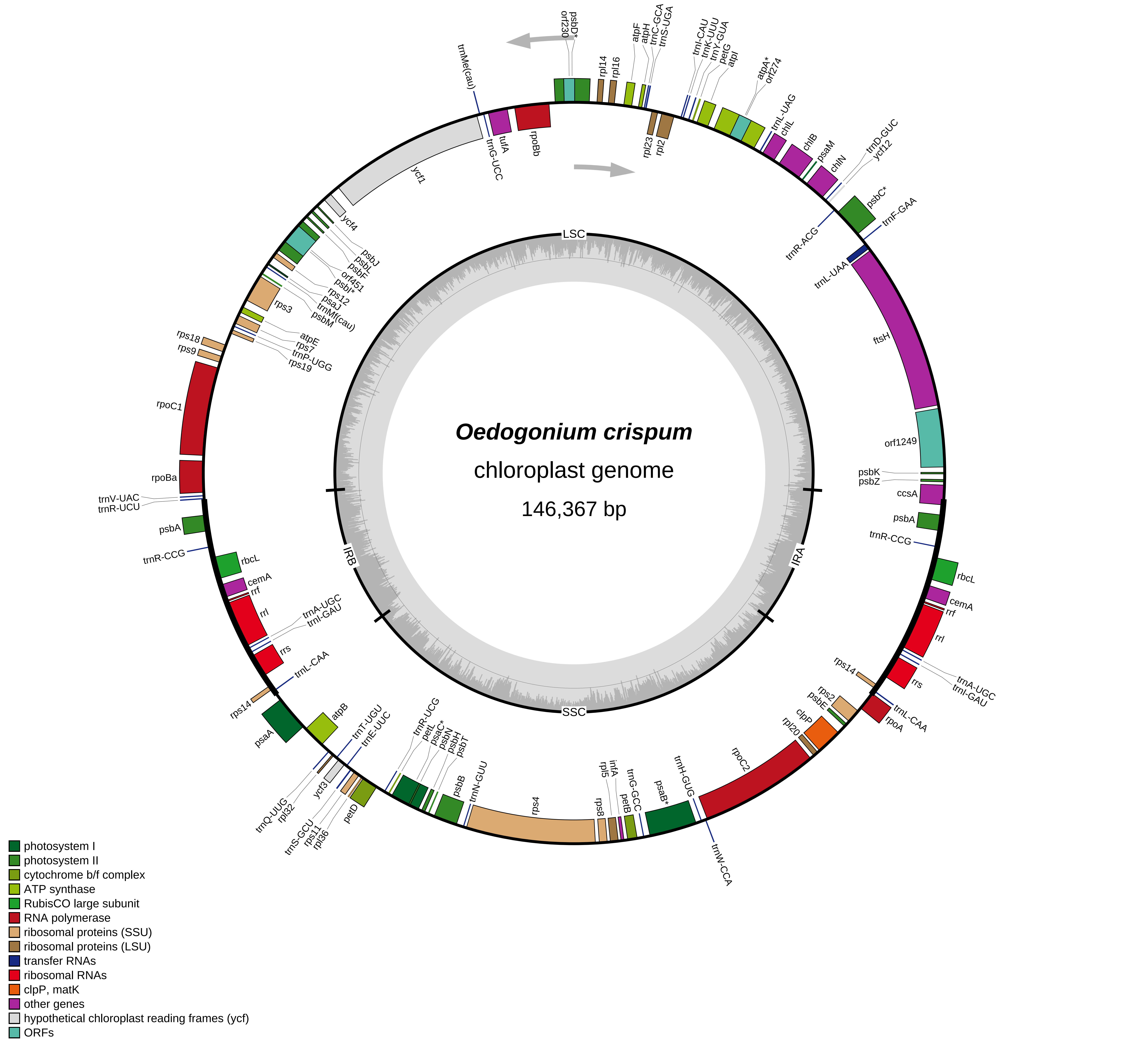

Oedogonium crispum
chloroplast genome
146,367 bp
LSC
IRA
SSC
IRB
photosystem I
photosystem II
cytochrome b/f complex
ATP synthase
RubisCO large subunit
RNA polymerase
ribosomal proteins (SSU)
ribosomal proteins (LSU)
transfer RNAs
ribosomal RNAs
clpP, matK
other genes
hypothetical chloroplast reading frames (ycf)
ORFs
psbD*
orf230
rpl14
rpl16
atpF
atpH
trnC-GCA
trnS-UGA
trnI-CAU
trnK-UUU
trnY-GUA
petG
atpI
atpA*
orf274
trnL-UAG
chlL
chlB
psaM
chlN
trnD-GUC
ycf12
psbC*
trnF-GAA
trnR-ACG
trnL-UAA
ftsH
orf1249
psbK
psbZ
ccsA
psbA
trnR-CCG
rbcL
cemA
rrf
rrl
trnA-UGC
trnI-GAU
rrs
rps14
trnL-CAA
rpoA
rps2
psbE
clpP
rpl20
rpoC2
trnH-GUG
trnW-CCA
psaB*
trnG-GCC
petB
infA
rpl5
rps8
rps4
trnN-GUU
psbB
trnR-UCG
petL
psaC*
psbN
psbH
psbT
trnT-UGU
trnE-UUC
atpB
petD
rps11
rpl36
trnS-GCU
ycf3
rpl32
trnQ-UUG
psaA
rps14
trnL-CAA
rrs
trnA-UGC
trnI-GAU
rrl
rrf
cemA
rbcL
trnR-CCG
psbA
trnV-UAC
trnR-UCU
rpoBa
rpoC1
rps18
rps9
atpE
rps7
trnP-UGG
rps19
rps3
rps12
psaJ
trnMf(cau)
psbM
orf451
psbI*
psbF
psbL
psbJ
ycf4
ycf1
trnMe(cau)
trnG-UCC
tufA
rpoBb
rpl23
rpl2
