# Supplementary material for: Chloroplast genomes of five Oedogonium species: genome structure, phylogenetic analysis and adaptive evolution
Source: BMC Genomics. 2021 Sep 30;22:707. doi: 10.1186/s12864-021-08006-1 (PMC8485540; doi:10.1186/s12864-021-08006-1)
Supplement: Supplementary file 5 — Additional file 5: Supplementary Fig. S5. Gene map of Oe. sp. (FACHB-3313) chloroplast genomes. Arrows show the direction of transcription. The same colour block shows the functional gene group (legend at bottom left). Transfer RNAs are represented by their one-letter amino acid code. The grey circle on the inside shows a graph of the GC content. [file 12864_2021_8006_MOESM5_ESM.pdf]

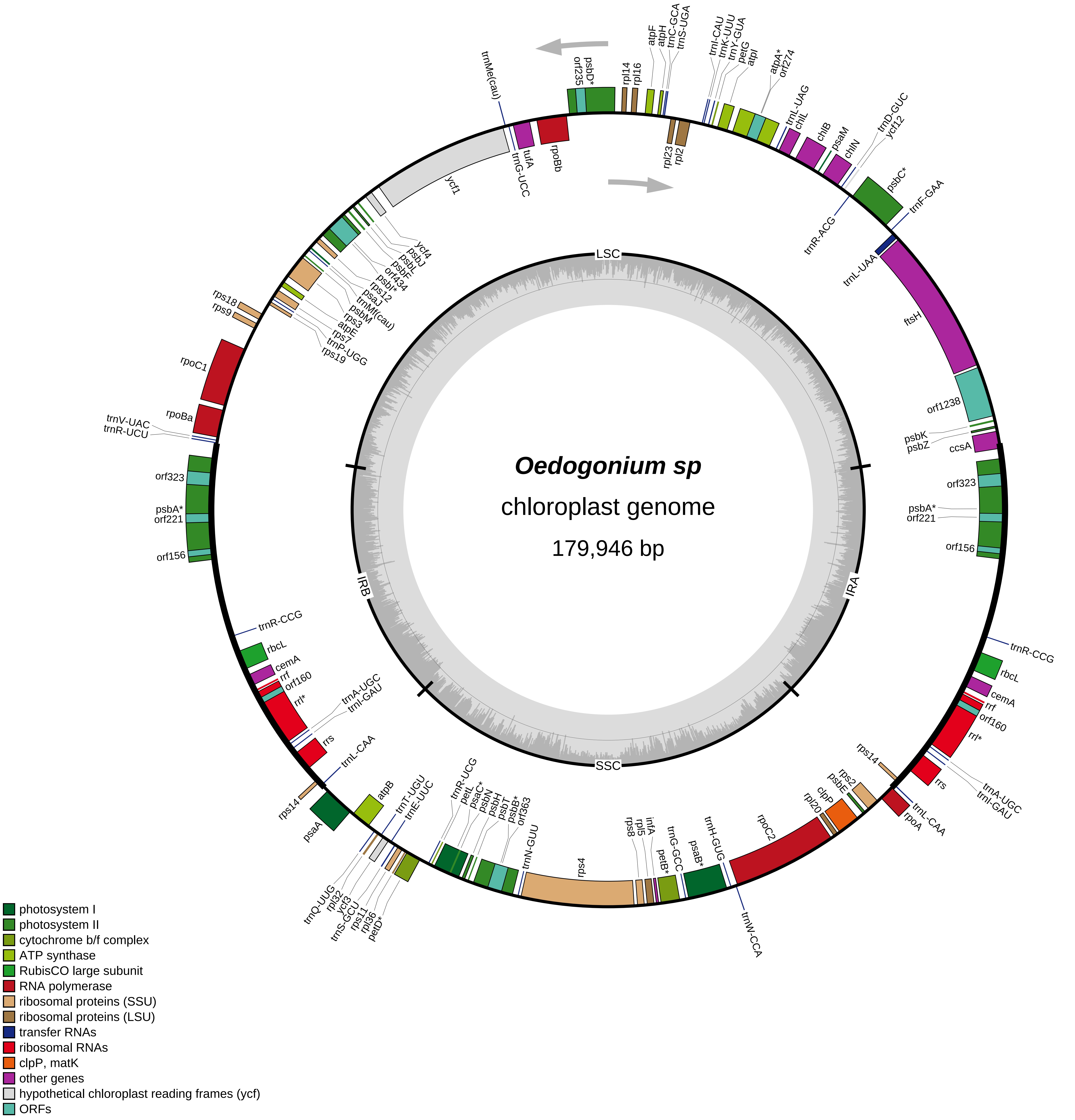

Oedogonium sp
chloroplast genome
179,946 bp
LSC
IRA
SSC
IRB
trnMe(cau)
orf235
psbD*
rpl14
rpl16
atpF
atpH
trnC-GCA
trnS-UGA
trnI-CAU
trnK-UUU
trnY-GUA
petG
atpI
atpA*
orf274
trnL-UAG
chlL
chlB
psaM
chlN
trnD-GUC
ycf12
psbC*
trnF-GAA
trnR-ACG
trnL-UAA
ftsH
orf1238
psbK
psbZ
ccsA
orf323
psbA*
orf221
orf156
trnR-CCG
rbcL
cemA
rrf
orf160
rrl*
trnA-UGC
trnI-GAU
rrs
trnL-CAA
rpoA
rps14
rps2
psbE
clpP
rpl20
rpoC2
trnW-CCA
trnH-GUG
psaB*
trnG-GCC
petB*
infA
rpl5
rps8
rps4
trnN-GUU
orf363
psbB*
psbT
psbH
psbN
psaC*
petL
trnR-UCG
trnE-UUC
trnT-UGU
atpB
psaA
trnQ-UUG
rpl32
ycf3
trnS-GCU
rps11
rpl36
petD*
rpoBa
trnV-UAC
trnR-UCU
rpoC1
rps18
rps9
rps19
trnP-UGG
rps7
atpE
rps3
psbM
trnMf(cau)
psaJ
rps12
psbI*
orf434
psbF
psbL
psbJ
ycf4
ycf1
trnG-UCC
tufA
rpoBb
rpl23
rpl2
photosystem I
photosystem II
cytochrome b/f complex
ATP synthase
RubisCO large subunit
RNA polymerase
ribosomal proteins (SSU)
ribosomal proteins (LSU)
transfer RNAs
ribosomal RNAs
clpP, matK
other genes
hypothetical chloroplast reading frames (ycf)
ORFs
