# Supplementary figures and images for: Chloroplast genomes of five Oedogonium species: genome structure, phylogenetic analysis and adaptive evolution
Source: BMC Genomics. 2021 Sep 30;22:707. doi: 10.1186/s12864-021-08006-1 (PMC8485540; doi:10.1186/s12864-021-08006-1)

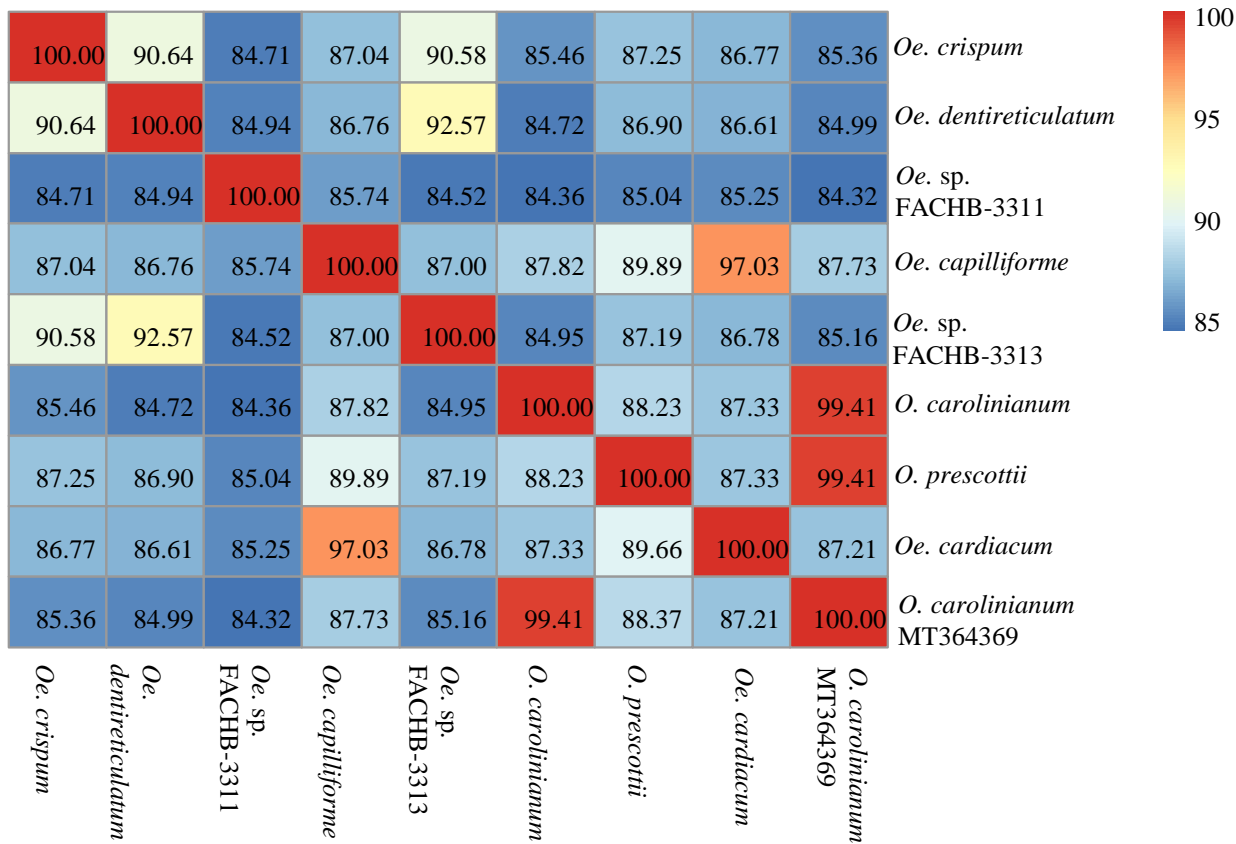

Supplement: Supplementary file 6 — Additional file 6: Supplementary Fig. S6. Heat map of ANI values of the nine Oedogoniales cp genomes. [file 12864_2021_8006_MOESM6_ESM.pdf]

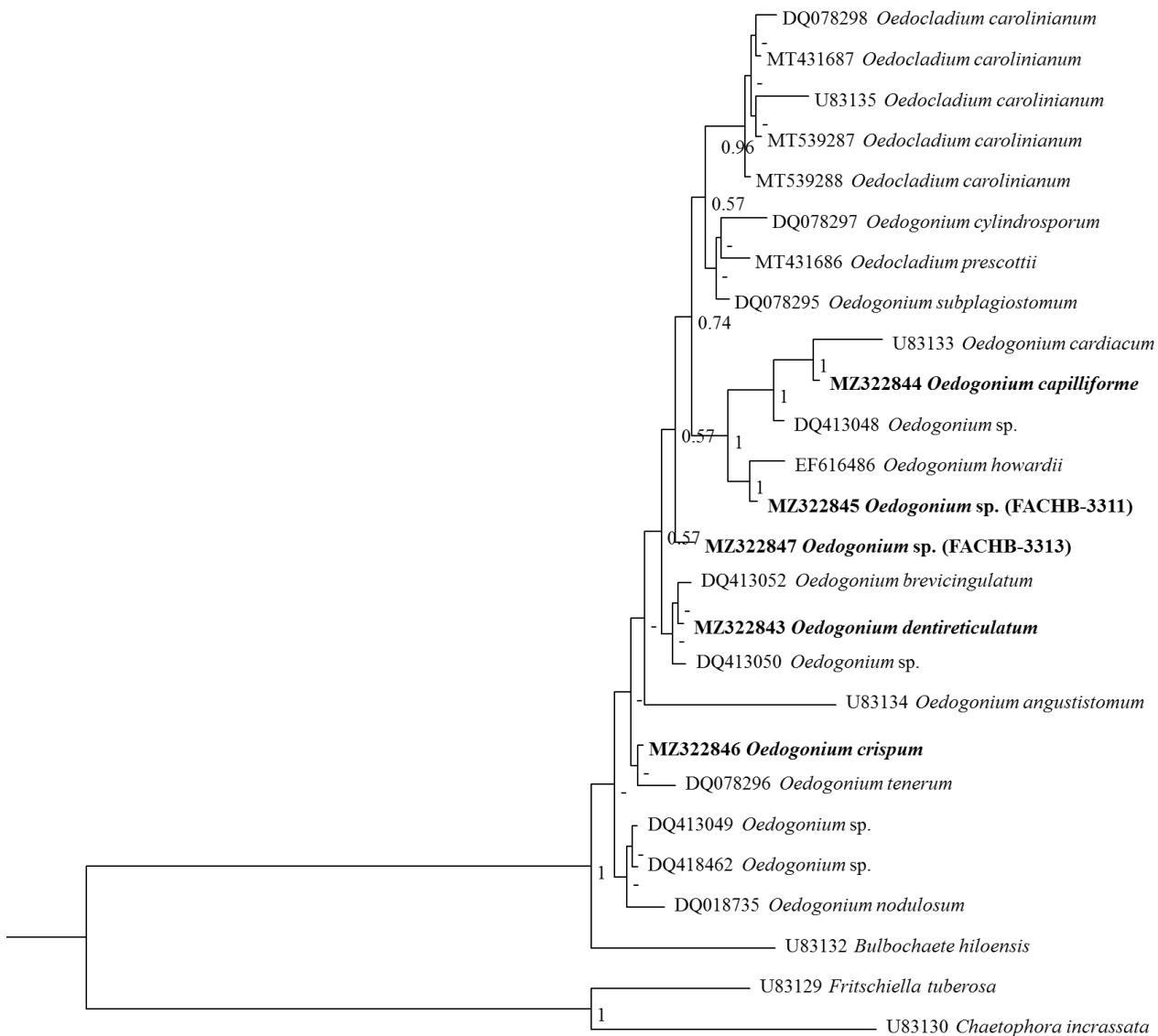

0.01

Supplement: Supplementary file 7 — Additional file 7: Supplementary Fig. S7. Phylogenetic tree of the Oedogoniales algae based on 18S rDNA sequences. Numbers at the branches represent Bayesian posterior probabilities (≥0.5). Branch lengths are proportional to the genetic distances, which are indicated by the scale bar (Bold species are the newly included in this study). [file 12864_2021_8006_MOESM7_ESM.pdf]
