# Supplementary material for: Chloroplast genomes of five Oedogonium species: genome structure, phylogenetic analysis and adaptive evolution
Source: BMC Genomics. 2021 Sep 30;22:707. doi: 10.1186/s12864-021-08006-1 (PMC8485540; doi:10.1186/s12864-021-08006-1)
Supplement: Supplementary file 8 — Additional file 8: Supplementary Table S1. Insertion sites of group I introns of the nine Oedogoniaes cp genomes [81]. [file 12864_2021_8006_MOESM8_ESM.docx]

**Supplementary information**

Supplementary table S1. Insertion sites of group I introns of the nine Oedogoniaes cp genomes.

|  |  | *Oe. dentireticulatum*  FACHB-3309 | *Oe. crispum*  FACHB-3310 | *Oe.* sp.  FACHB-3311 | *Oe. capilliforme*  FACHB-3312 | *Oe.* sp.  FACHB-3313 | *Oe*. *cardiacum* | *O. prescottii* | *O. carolinianum* | *O. carolinianum*  MT364369 |
| --- | --- | --- | --- | --- | --- | --- | --- | --- | --- | --- |
| *atp*A | 485 |  |  | — |  |  |  |  |  |  |
| *atp*A | 489 |  |  |  | — | — | — |  | — | — |
| *atp*A | 504 |  | — |  |  |  |  |  |  |  |
| *atp*I | 625 |  |  |  |  |  |  |  | — |  |
| *psa*B | 1771 |  |  | — |  |  |  |  |  |  |
| *psb*A | 179 |  |  |  |  |  | — |  |  |  |
| *psb*A | 181 | — |  | — |  | — |  |  |  |  |
| *psb*A | 275 | — |  |  |  |  |  |  |  |  |
| *psb*A | 277 |  |  |  |  | — | — |  |  |  |
| *psb*A | 278 |  |  | — |  |  |  |  |  |  |
| *psb*A | 385 |  |  |  |  | — | — |  |  |  |
| *psb*A | 409 |  |  | — |  |  | — |  |  |  |
| *psb*A | 413 | *—* |  |  |  |  |  |  |  |  |
| *psb*A | 415 |  |  |  |  | — |  |  |  |  |
| *psb*A | 526 | — |  | — | — |  | — |  |  |  |
| *psb*A | 548 |  |  |  |  |  |  |  | — | — |
| *psb*A | 549 |  |  |  |  |  | — |  |  |  |
| *psb*A | 550 |  |  |  | — | — |  |  |  |  |
| *psb*A | 560 | — |  |  |  |  |  |  |  |  |
| *psb*A | 570 |  |  |  |  |  |  |  | — |  |
| *psb*A | 571 |  |  |  | — | — | — |  |  |  |
| *psb*A | 573 |  |  | — |  |  |  |  |  |  |
| *psb*A | 646 | — |  |  | — | — | — |  |  |  |
| *psb*A | 652 |  |  | — |  |  |  |  |  |  |
| *psb*A | 757 |  |  |  |  |  | — |  |  |  |
| *psb*A | 848 |  |  | — |  |  |  |  |  |  |
| *psb*A | 892 |  |  |  |  | — |  |  |  |  |
| *psb*A | 898 |  |  |  |  |  | — |  |  |  |
| *psb*A | 900 |  |  | — |  |  |  |  |  |  |
| *psb*A | 901 | — |  |  | — |  |  |  |  |  |
| *psb*C | 300 |  |  | — |  |  |  |  |  |  |
| *psb*C | 579 |  | — | — | — | — | — |  | — | — |
| *psb*C | 708 |  |  |  |  |  |  |  | — | — |
| *psb*C | 918 |  |  | — |  | — |  |  |  |  |
| *psb*C | 1009 |  |  |  | — | — | — |  |  |  |
| *psb*D | 553 |  |  |  |  | — |  |  |  |  |
| *psb*D | 746 |  |  |  |  |  |  |  | — |  |
| *psb*D | 748 | — | — | — |  | — |  |  |  |  |
| rrl | 325 |  |  | — |  |  |  |  |  |  |
| rrl | 326 |  |  |  |  | — |  |  |  |  |
| rrl | 1936 |  |  |  |  |  | — |  |  |  |
| rrl | 2454 |  |  | — |  | — |  |  |  |  |
| rrl | 2505 |  |  |  | — |  |  |  |  |  |
| rrl | 2598 | — |  |  |  | — |  |  |  |  |
| rrl | 2601 |  |  | — | — |  | — |  |  |  |
| rrs | 427 |  |  |  | — |  | — |  |  |  |
| *trn*L(uaa) | 35 | — | — | — | — | — | — | — | — | — |

^a^ Group I Intron insertion sites in protein-coding and tRNA genes are given relative to the corresponding genes in the deeply-diverging streptophyte alga *Mesostigma viride* [75]; insertion sites in rrs and rrl are given relative to *Escherichia coli* 16S and 23S rRNAs, respectively. For each insertion site, the position corresponding to the nucleotide immediately preceding the intron is reported.
