# Supplementary material for: Chloroplast genomes of five Oedogonium species: genome structure, phylogenetic analysis and adaptive evolution
Source: BMC Genomics. 2021 Sep 30;22:707. doi: 10.1186/s12864-021-08006-1 (PMC8485540; doi:10.1186/s12864-021-08006-1)
Supplement: Supplementary file 9 — Additional file 9: Supplementary Table S2. Insertion sites of group II introns of the nine Oedogoniaes cp genomes [81]. [file 12864_2021_8006_MOESM9_ESM.docx]

Supplementary table S2. Insertion sites of group II introns of the nine Oedogoniaes cp genomes.

|  |  | *Oe. dentireticulatum*  FACHB-3309 | *Oe.crispum*  FACHB-3310 | *Oe.*sp.  FACHB-3311 | *Oe. capilliforme*  FACHB-3312 | *Oe.*sp.  FACHB-3313 | *Oe*. *cardiacum* | *O. prescottii* | *O. carolinianum* | *O. carolinianum*  MT364369 |
| --- | --- | --- | --- | --- | --- | --- | --- | --- | --- | --- |
| *atp*A | 103 |  |  | — |  |  |  |  |  |  |
| *atp*A | 748 |  |  | — |  |  |  |  |  |  |
| *atp*A | 669 |  |  |  |  |  |  |  | — | — |
| *atp*B | 688 |  |  |  |  |  |  | — |  |  |
| *atp*I | 162 |  |  | — |  |  |  |  |  |  |
| *atp*I | 626 |  |  | — |  |  |  |  |  |  |
| *atp*I | 625 |  |  |  |  |  |  |  |  | — |
| *chl*B | 76 |  |  | — |  |  |  |  |  |  |
| *chl*L | 409 |  |  | — |  |  |  |  |  |  |
| *pet*B | 415 |  |  |  |  | — |  |  | — | — |
| *pet*D | 4 | — | — | — | — | — | — | — | — | — |
| *psa*A | 1794 |  |  | — |  |  |  |  | — | — |
| *psa*B | 1771 | — | — |  |  | — |  |  |  |  |
| *psa*B | 562 |  |  |  |  |  |  |  | — | — |
| *psa*C | 25 | — | — | — | — | — | — | — | — | — |
| *psb*B | 601 |  |  |  |  | — |  |  |  |  |
| *psb*B | 148 |  |  |  | — |  | — | — | — | — |
| *psb*C | 885 |  |  | — |  |  |  |  |  |  |
| *psb*C | 1088 |  |  |  |  |  |  |  | — | — |
| *psb*I | 22 | — | — |  | — | — | — | — | — | — |

^a^ Group II Intron insertion sites in protein-coding and tRNA genes are given relative to the corresponding genes in the deeply-diverging streptophyte alga *Mesostigma viride* [75]; insertion sites in rrs and rrl are given relative to *Escherichia coli* 16S and 23S rRNAs, respectively. For each insertion site, the position corresponding to the nucleotide immediately preceding the intron is reported.
