# Supplementary material for: Chloroplast genomes of five Oedogonium species: genome structure, phylogenetic analysis and adaptive evolution
Source: BMC Genomics. 2021 Sep 30;22:707. doi: 10.1186/s12864-021-08006-1 (PMC8485540; doi:10.1186/s12864-021-08006-1)
Supplement: Supplementary file 10 — Additional file 10: Supplementary Table S3. Substitution rates in the chloroplast protein coding genes of Oedogoniales species. [file 12864_2021_8006_MOESM10_ESM.docx]

Supplementary table S3. Substitution rates in the chloroplast protein coding genes of Oedogoniales species.

| Genes | Nonsynonymous substitution rate (dN) | | | | Genes | Synonymous substitution rate (dS) | | | |
| --- | --- | --- | --- | --- | --- | --- | --- | --- | --- |
|  | terrestrial  species | aquatic  species | *P* value | FDR |  | terrestrial  species | aquatic  species | *P* value | FDR |
| atpA | 0.0077 | 0.0094 | 0.5334 | 0.8229 | atpA | 0.6553 | 0.6931 | 1.0000 | 1.0000 |
| atpB | 0.0077 | 0.0084 | 0.6198 | 0.8740 | atpB | 0.6762 | 0.7187 | 1.0000 | 1.0000 |
| atpE | 0.0318 | 0.0320 | 1.0000 | 1.0000 | atpE | 0.7482 | 0.7040 | 0.4606 | 0.9565 |
| atpF | 0.0221 | 0.0241 | 0.7122 | 0.8740 | atpF | 0.6989 | 0.7487 | 0.7122 | 0.9971 |
| atpH | 0.0247 | 0.0187 | 0.2663 | 0.6848 | atpH | 0.2758 | 0.2746 | 0.7110 | 0.9971 |
| atpI | 0.0245 | 0.0208 | 1.0000 | 1.0000 | atpI | 0.6375 | 0.7341 | 0.2683 | 0.9565 |
| cemA | 0.0107 | 0.0125 | 0.7122 | 0.8740 | cemA | 0.1291 | 0.1738 | 0.5386 | 0.9971 |
| chlB | 0.0137 | 0.0167 | 0.3893 | 0.7508 | chlB | 0.8127 | 0.8693 | 0.9021 | 0.9971 |
| chlL | 0.0118 | 0.0156 | 0.1761 | 0.6792 | chlL | 1.0688 | 1.1798 | 0.9021 | 0.9971 |
| chlN | 0.0202 | 0.0217 | 0.4127 | 0.7685 | chlN | 0.7812 | 0.9273 | 0.2857 | 0.9565 |
| clpP | 0.0439 | 0.0507 | 1.0000 | 1.0000 | clpP | 1.1387 | 1.3282 | 0.4127 | 0.9565 |
| petB | 0.0013 | 0.0023 | 0.2249 | 0.6848 | petB | 0.5057 | 0.5985 | 0.1761 | 0.9509 |
| petD | 0.0062 | 0.0088 | 0.1039 | 0.5393 | petD | 0.5301 | 0.6733 | 0.0651 | 0.9509 |
| petG | 0.0002 | 0.0002 | 0.5186 | 0.8229 | petG | 0.2367 | 0.2485 | 0.4407 | 0.9565 |
| petL | 0.0110 | 0.0165 | 0.0189 | 0.3862 | petL | 2.4752 | 3.3188 | 0.9021 | 0.9971 |
| psaA | 0.0035 | 0.0044 | 0.0606 | 0.4898 | psaA | 0.6233 | 0.6738 | 0.5556 | 0.9971 |
| psaB | 0.0051 | 0.0060 | 0.0358 | 0.3862 | psaB | 0.5862 | 0.6690 | 0.4127 | 0.9565 |
| psaC | 0.0051 | 0.0060 | 0.0358 | 0.3862 | psaC | 0.5862 | 0.6690 | 0.4127 | 0.9565 |
| psaJ | 0.0050 | 0.0107 | 0.3706 | 0.7508 | psaJ | 0.4497 | 1.0217 | 0.1761 | 0.9509 |
| psbA | 0.0085 | 0.0095 | 0.5556 | 0.8333 | psbA | 0.1381 | 0.1366 | 1.0000 | 1.0000 |
| psbB | 0.0070 | 0.0081 | 0.6168 | 0.8740 | psbB | 0.5347 | 0.5670 | 0.7302 | 0.9971 |
| psbD | 0.0028 | 0.0035 | 0.2602 | 0.6848 | psbD | 0.3367 | 0.3438 | 0.9048 | 0.9971 |
| psbE | 0.0039 | 0.0019 | 0.2475 | 0.6848 | psbE | 0.4228 | 0.5557 | 0.3893 | 0.9565 |
| psbF | 0.0061 | 0.0068 | 1.0000 | 1.0000 | psbF | 0.1406 | 0.1651 | 0.9017 | 0.9971 |
| psbH | 0.0063 | 0.0071 | 0.5224 | 0.8229 | psbH | 0.4224 | 0.6300 | 0.0365 | 0.9509 |
| psbI | 0.0013 | 0.0031 | 0.2404 | 0.6848 | psbI | 0.0956 | 0.1174 | 1.0000 | 1.0000 |
| psbJ | 0.0082 | 0.0105 | 0.6999 | 0.8740 | psbJ | 0.0537 | 0.0855 | 0.1099 | 0.9509 |
| psbK | 0.0071 | 0.0118 | 0.3231 | 0.7270 | psbK | 0.9073 | 1.3213 | 1.0000 | 1.0000 |
| psbL | 0.0134 | 0.0171 | 0.1084 | 0.5393 | psbL | 0.2106 | 0.2638 | 0.1761 | 0.9509 |
| psbM | 0.0069 | 0.0032 | 0.1039 | 0.5393 | psbM | 0.2378 | 0.2378 | 0.7110 | 0.9971 |
| psbN | 0.0060 | 0.0084 | 0.0342 | 0.3862 | psbN | 4.5117 | 3.3457 | 0.4127 | 0.9565 |
| psbT | 0.0049 | 0.0044 | 1.0000 | 1.0000 | psbT | 0.1250 | 0.1607 | 0.4509 | 0.9565 |
| psbZ | 0.0136 | 0.0147 | 1.0000 | 1.0000 | psbZ | 1.1733 | 1.9467 | 0.2683 | 0.9565 |
| rpl14 | 0.0021 | 0.0026 | 0.2089 | 0.6848 | rpl14 | 0.6933 | 0.8295 | 0.2683 | 0.9565 |
| rpl16 | 0.0076 | 0.0070 | 0.5186 | 0.8229 | rpl16 | 0.4673 | 0.5320 | 0.5386 | 0.9971 |
| rpl2 | 0.0089 | 0.0112 | 1.0000 | 1.0000 | rpl2 | 0.7247 | 0.8329 | 0.1761 | 0.9509 |
| rpl20 | 0.0280 | 0.0296 | 0.7122 | 0.8740 | rpl20 | 0.9998 | 1.2338 | 0.1761 | 0.9509 |
| rpl23 | 0.0316 | 0.0366 | 0.2857 | 0.7013 | rpl23 | 1.4928 | 1.5631 | 0.2857 | 0.9565 |
| rpl36 | 0.0020 | 0.0037 | 0.3169 | 0.7270 | rpl36 | 0.8097 | 0.9989 | 0.9021 | 0.9971 |
| rpl5 | 0.0087 | 0.0106 | 0.3873 | 0.7508 | rpl5 | 1.2085 | 1.6081 | 0.1099 | 0.9509 |
| rps11 | 0.0029 | 0.0039 | 0.1761 | 0.6792 | rps11 | 1.6487 | 1.6226 | 0.7302 | 0.9971 |
| rps12 | 0.0084 | 0.0082 | 1.0000 | 1.0000 | rps12 | 0.7467 | 1.0558 | 0.0195 | 0.9509 |
| rps14 | 0.0033 | 0.0071 | 0.0237 | 0.3862 | rps14 | 0.1471 | 0.1762 | 0.3211 | 0.9565 |
| rps18 | 0.0163 | 0.0175 | 0.9009 | 1.0000 | rps18 | 0.8445 | 0.9232 | 0.9048 | 0.9971 |
| rps19 | 0.0152 | 0.0143 | 0.4568 | 0.8222 | rps19 | 1.4906 | 1.3254 | 0.9021 | 0.9971 |
| rps2 | 0.0170 | 0.0222 | 0.1099 | 0.5393 | rps2 | 1.5645 | 1.4131 | 0.9021 | 0.9971 |
| rps3 | 0.0945 | 0.1082 | 0.2187 | 0.6848 | rps3 | 1.1119 | 1.3369 | 0.4127 | 0.9565 |
| rps7 | 0.0291 | 0.0361 | 0.1761 | 0.6792 | rps7 | 1.1878 | 1.3427 | 0.3893 | 0.9565 |
| rps8 | 0.0312 | 0.0359 | 0.0635 | 0.4898 | rps8 | 0.9556 | 1.0581 | 0.7302 | 0.9971 |
| rps9 | 0.0188 | 0.0223 | 0.3832 | 0.7508 | rps9 | 1.1565 | 1.2341 | 0.9021 | 0.9971 |
| tufA | 0.0128 | 0.0137 | 0.9021 | 1.0000 | tufA | 0.6846 | 0.7696 | 0.9021 | 0.9971 |
| ycf12 | 0.0555 | 0.0552 | 0.7122 | 0.8740 | ycf12 | 0.4076 | 0.4137 | 0.9021 | 0.9971 |
| ycf3 | 0.0026 | 0.0032 | 0.5334 | 0.8229 | ycf3 | 0.9802 | 1.1891 | 0.9048 | 0.9971 |
| ycf4 | 0.0102 | 0.0129 | 0.7122 | 0.8740 | ycf4 | 0.8823 | 0.9800 | 0.5386 | 0.9971 |

Benjamini, Y., and Hochberg, Y. (1995). Controlling the false discovery rate: a

practical and powerful approach to multiple testing. J. R. Stat. Soc. Ser. B Stat.

57, 289–300.
