# Supplementary material for: Chloroplast genomes of five Oedogonium species: genome structure, phylogenetic analysis and adaptive evolution
Source: BMC Genomics. 2021 Sep 30;22:707. doi: 10.1186/s12864-021-08006-1 (PMC8485540; doi:10.1186/s12864-021-08006-1)
Supplement: Supplementary file 1 — Additional file 1: Supplementary Fig. S1. Gene map of Oe. dentireticulatum chloroplast genomes. [file 12864_2021_8006_MOESM1_ESM.pdf]

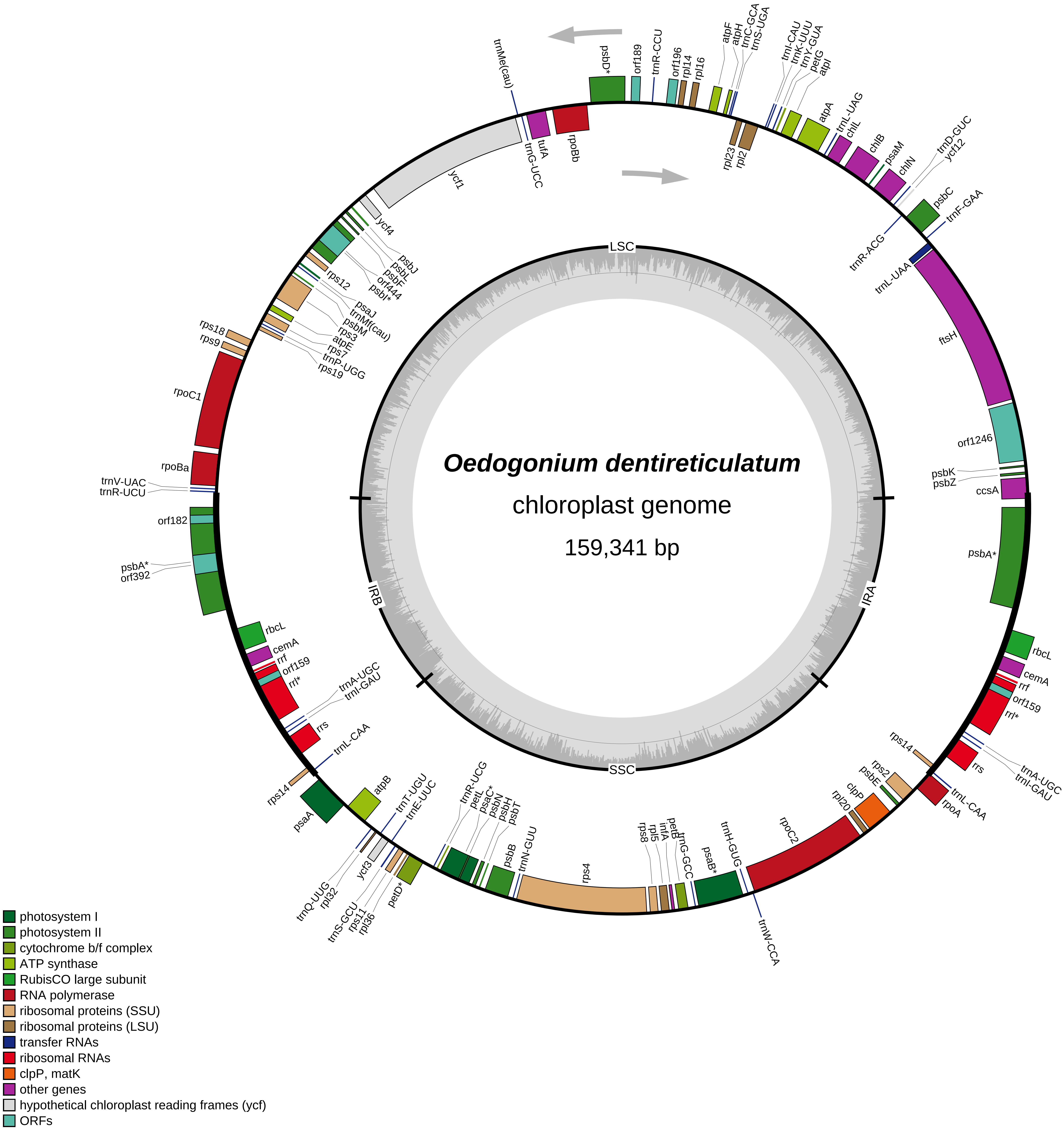

Oedogonium dentireticulatum
chloroplast genome
159,341 bp
LSC
IRA
SSC
IRB
psbD*
orf189
trnR-CCU
orf196
rpl14
rpl16
atpF
atpH
trnC-GCA
trnS-UGA
trnI-CAU
trnK-UUU
trnY-GUA
petG
atpI
atpA
trnL-UAG
chlL
chlB
psaM
chlN
trnD-GUC
ycf12
psbC
trnF-GAA
trnR-ACG
trnL-UAA
ftsH
orf1246
psbK
psbZ
ccsA
psbA*
rbcL
cemA
rrf
orf159
rrl*
rrs
rps14
trnA-UGC
trnI-GAU
trnL-CAA
rpoA
rps2
psbE
clpP
rpl20
rpoC2
trnW-CCA
trnH-GUG
psaB*
trnG-GCC
petB
infA
rpl5
rps8
rps4
trnN-GUU
psbB
psbT
psbH
psbN
psaC*
petL
trnR-UCG
petD*
rpl36
rps11
trnS-GCU
ycf3
rpl32
trnQ-UUG
trnE-UUC
trnT-UGU
atpB
psaA
rps14
trnL-CAA
rrs
trnA-UGC
trnI-GAU
rrl*
orf159
rrf
cemA
rbcL
psbA*
orf392
orf182
trnV-UAC
trnR-UCU
rpoBa
rpoC1
rps18
rps9
rps19
trnP-UGG
rps7
atpE
rps3
psbM
trnMf(cau)
psaJ
rps12
psbI*
orf444
psbF
psbL
psbJ
ycf4
ycf1
trnG-UCC
tufA
rpoBb
trnMe(cau)
rpl23
rpl2
photosystem I
photosystem II
cytochrome b/f complex
ATP synthase
RubisCO large subunit
RNA polymerase
ribosomal proteins (SSU)
ribosomal proteins (LSU)
transfer RNAs
ribosomal RNAs
clpP, matK
other genes
hypothetical chloroplast reading frames (ycf)
ORFs
