# Supplementary material for: Chloroplast genomes of five Oedogonium species: genome structure, phylogenetic analysis and adaptive evolution
Source: BMC Genomics. 2021 Sep 30;22:707. doi: 10.1186/s12864-021-08006-1 (PMC8485540; doi:10.1186/s12864-021-08006-1)
Supplement: Supplementary file 3 — Additional file 3: Supplementary Fig. S3. Gene map of Oe. sp. (FACHB-3311) chloroplast genomes. [file 12864_2021_8006_MOESM3_ESM.pdf]

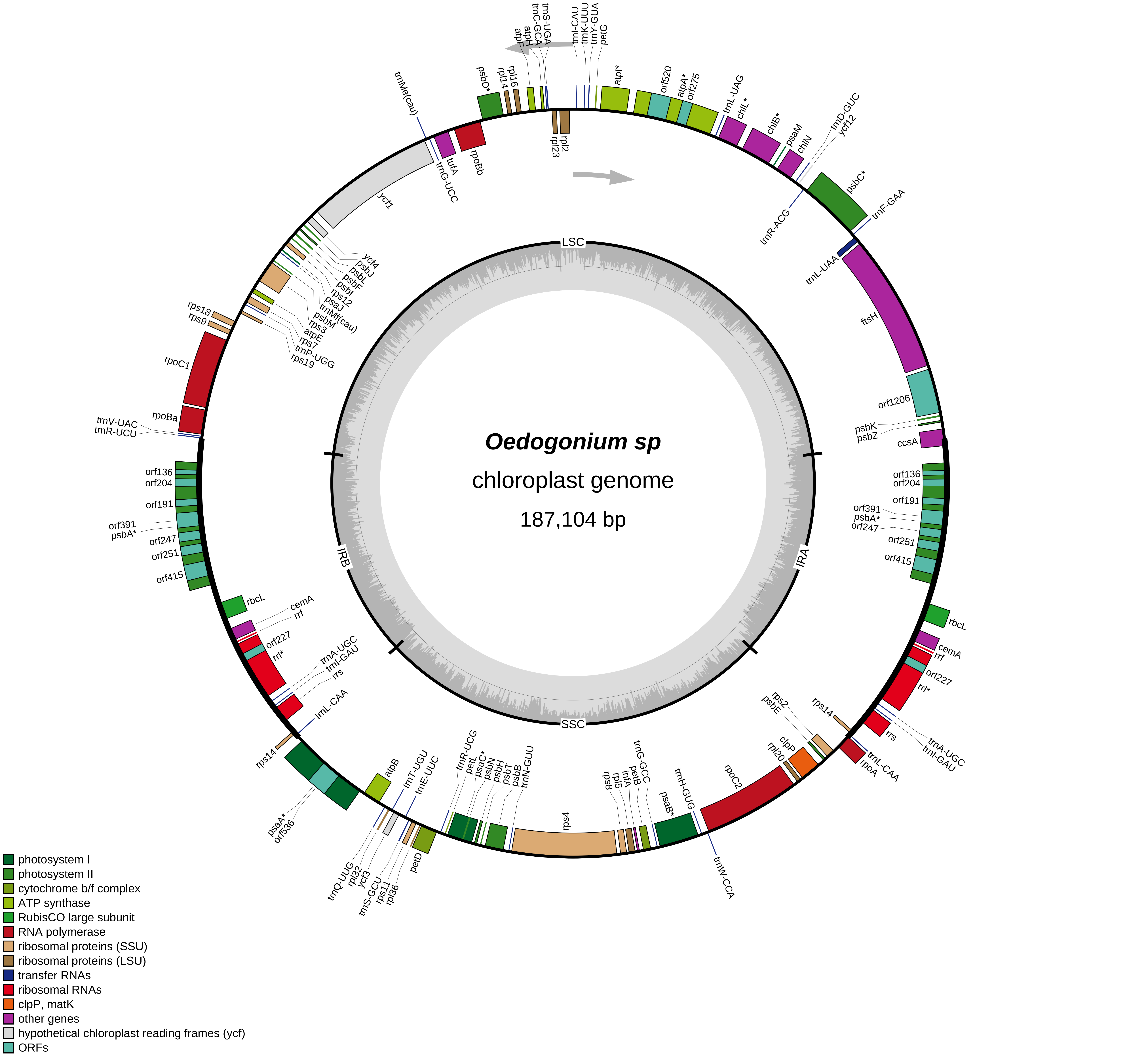

Oedogonium sp
chloroplast genome
187,104 bp
LSC
IRA
SSC
IRB
trnMe(cau)
psbD*
rpl14
rpl16
atpF
atpH
trnC-GCA
trnS-UGA
trnI-CAU
trnK-UUU
trnY-GUA
petG
atpI*
orf520
atpA*
orf275
trnL-UAG
chlL *
chlB*
psaM
chlN
trnD-GUC
ycf12
psbC*
trnF-GAA
trnR-ACG
trnL-UAA
ftsH
orf1206
psbK
psbZ
ccsA
orf136
orf204
orf191
orf391
psbA*
orf247
orf251
orf415
rbcL
cemA
rrf
orf227
rrl*
rrs
trnA-UGC
trnI-GAU
trnL-CAA
rpoA
rps14
rps2
psbE
clpP
rpl20
rpoC2
trnH-GUG
trnW-CCA
psaB*
trnG-GCC
petB
infA
rpl5
rps8
rps4
trnN-GUU
psbB
psbT
psbH
psbN
psaC*
petL
trnR-UCG
petD
trnE-UUC
trnT-UGU
atpB
rpl36
rps11
trnS-GCU
ycf3
rpl32
trnQ-UUG
psaA*
orf536
rpoBa
trnV-UAC
trnR-UCU
rpoC1
rps18
rps9
rps19
trnP-UGG
rps7
atpE
rps3
psbM
trnMf(cau)
psaJ
rps12
psbI
psbF
psbL
psbJ
ycf4
ycf1
trnG-UCC
tufA
rpoBb
rpl2
rpl23
photosystem I
photosystem II
cytochrome b/f complex
ATP synthase
RubisCO large subunit
RNA polymerase
ribosomal proteins (SSU)
ribosomal proteins (LSU)
transfer RNAs
ribosomal RNAs
clpP, matK
other genes
hypothetical chloroplast reading frames (ycf)
ORFs
