# Supplementary material for: Chloroplast genomes of five Oedogonium species: genome structure, phylogenetic analysis and adaptive evolution
Source: BMC Genomics. 2021 Sep 30;22:707. doi: 10.1186/s12864-021-08006-1 (PMC8485540; doi:10.1186/s12864-021-08006-1)
Supplement: Supplementary file 4 — Additional file 4: Supplementary Fig. S4. Gene map of Oe. capilliforme chloroplast genomes. [file 12864_2021_8006_MOESM4_ESM.pdf]

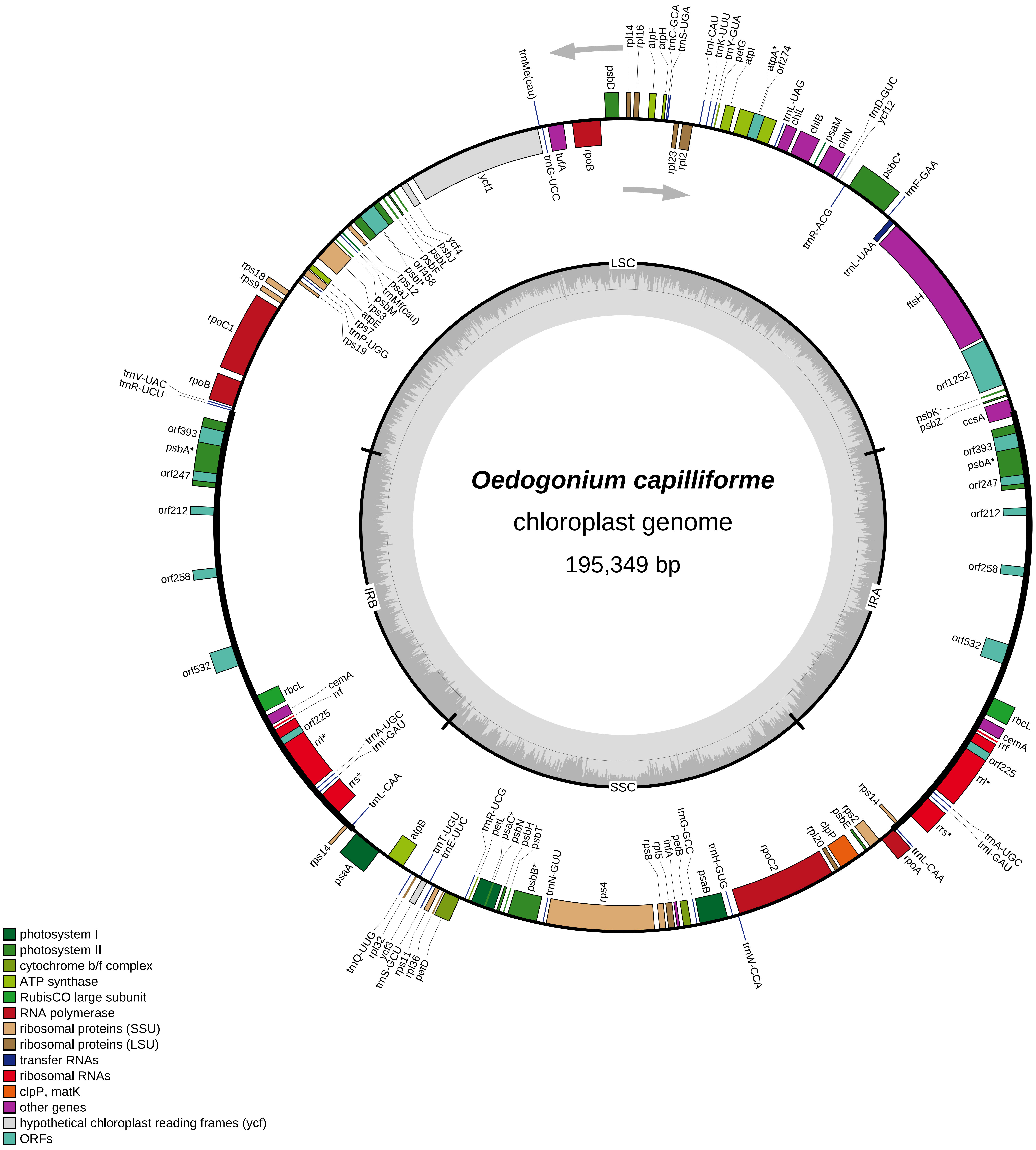
Oedogonium capilliforme
chloroplast genome
195,349 bp
LSC
SSC
IRA
IRB
psbD
rpl14
rpl16
atpF
atpH
trnC-GCA
trnS-UGA
trnI-CAU
trnK-UUU
trnY-GUA
petG
atpI
atpA*
orf274
trnL-UAG
chlL
chlB
psaM
chlN
trnD-GUC
ycf12
psbC*
trnF-GAA
trnR-ACG
trnL-UAA
ftsH
orf1252
psbK
psbZ
ccsA
orf393
psbA*
orf247
orf212
orf258
orf532
rbcL
cemA
rrf
orf225
rrl*
rrs*
trnA-UGC
trnI-GAU
trnL-CAA
rps14
rpoA
rps2
psbE
clpP
rpl20
rpoC2
trnW-CCA
trnH-GUG
psaB
trnG-GCC
petB
infA
rpl5
rps8
rps4
trnN-GUU
psbB*
psbT
psbH
psbN
psaC*
petL
trnR-UCG
trnT-UGU
trnE-UUC
atpB
psaA
trnQ-UUG
rpl32
ycf3
trnS-GCU
rps11
rpl36
petD
trnMe(cau)
rpl23
rpl2
rpoB
tufA
trnG-UCC
ycf1
ycf4
psbJ
psbL
psbF
orf458
psbI*
rps12
psaJ
trnMf(cau)
psbM
rps3
atpE
rps7
trnP-UGG
rps19
rps18
rps9
rpoC1
trnV-UAC
trnR-UCU
photosystem I
photosystem II
cytochrome b/f complex
ATP synthase
RubisCO large subunit
RNA polymerase
ribosomal proteins (SSU)
ribosomal proteins (LSU)
transfer RNAs
ribosomal RNAs
clpP, matK
other genes
hypothetical chloroplast reading frames (ycf)
ORFs
